# MTOR maintains endothelial cell integrity to limit lung vascular injury

**DOI:** 10.1016/j.jbc.2024.107952

**Published:** 2024-11-06

**Authors:** Michelle Warren Millar, Rauf A. Najar, Spencer A. Slavin, Mohammad Shadab, Imran Tahir, Zahra Mahamed, Xin Lin, Jun-ichi Abe, Terry W. Wright, David A. Dean, Fabeha Fazal, Arshad Rahman

**Affiliations:** 1 Department of Pediatrics, University of Rochester School of Medicine and Dentistry, Rochester, New York, USA; 2 Department of Cardiology, The University of Texas MD Anderson Cancer Center, Houston, Texas, USA

**Keywords:** endothelial cells, lung vascular injury, MTOR, gene therapy, VE-cadherin, VEGFR2

## Abstract

The functional and structural integrity of the endothelium is essential for vascular homeostasis. Loss of barrier function in quiescent and migratory capacity in proliferative endothelium causes exuberant vascular permeability, a cardinal feature of many inflammatory diseases including acute lung injury (ALI). However, the signals governing these fundamental endothelial cell (EC) functions are poorly understood. Here, we identify mechanistic target of rapamycin (MTOR) as an important link in preserving the barrier integrity and migratory/angiogenic responses in EC and preventing lung vascular injury and mortality in mice. Knockdown of *MTOR* in EC altered cell morphology, impaired proliferation and migration, and increased endocytosis of cell surface vascular endothelial (VE)-cadherin leading to disrupted barrier function. MTOR-depleted EC also exhibited reduced VE-cadherin and vascular endothelial growth factor receptor-2 (VEGFR2) levels mediated in part by autophagy. Similarly, lungs from mice with EC-specific MTOR deficiency displayed spontaneous vascular leakage marked by decreased VE-cadherin and VEGFR2 levels, indicating that MTOR deficiency in EC is sufficient to disrupt lung vascular integrity and may be a key pathogenic mechanism of ALI. Indeed, MTOR as well as VEGFR2 and VE-cadherin levels were markedly reduced in injured mouse lungs or EC. Importantly, EC-targeted gene transfer of MTOR complementary DNA, either prophylactically or therapeutically, mitigated inflammatory lung injury, and improved lung function and survival in mouse models of ALI. These findings reveal an essential role of MTOR in maintaining EC function, identify loss of endothelial MTOR as a key mechanism of lung vascular injury, and show the therapeutic potential of EC-targeted MTOR expression in combating ALI and mortality in mice.

Acute lung injury (ALI) and its more severe form acute respiratory distress syndrome (ARDS) are common causes of respiratory failure in critically ill patients, and most current therapies rely on supportive care with no effective therapeutic options available to improve the clinical outcome (1, 2, 3, 4). Endothelial cell (EC) dysfunction is a prominent feature of many inflammatory disease states including ALI/ARDS (5). EC line the blood vessels of major organs and represent the first barrier between blood and the surrounding tissue, and vascular integrity is essential for maintaining homeostasis (6). Biological, chemical, and mechanical insults can cause EC to lose their barrier function and express proinflammatory mediators (cytokines and chemokines), leading to vascular permeability and inflammatory cell recruitment in the lung (7, 8). Adherens junctions (AJs) are major determinants of vascular barrier integrity and are formed by homophilic interactions of cell surface vascular endothelial (VE)-cadherin on adjacent EC (9, 10). Intact AJs allow for selective passage of fluid and small molecules from the blood into the interstitial space. AJ disruption can occur by loss of VE-cadherin interactions secondary to downregulation of VE-cadherin expression, or removal from the cell surface by endocytosis and degradation of VE-cadherin through phosphorylation- and ubiquitination-dependent mechanisms (11, 12, 13, 14, 15, 16). The resulting AJ disassembly causes vascular permeability, which further facilitates immune cell transmigration from circulation (11, 14, 17). The coordinated actions of barrier disruption and inflammation contribute to the progression of ALI. While maintaining an intact barrier in quiescent endothelium is essential for vascular integrity, ECs, in their subconfluent state or after injury, have unique angiogenic properties that allow them to proliferate and migrate to form new blood vessels or restore barrier function after injury (18, 19). These new vessels enhance blood supply and nutrient delivery to wounded areas and facilitate healing (18). The angiogenic activity of EC is governed in major part by vascular endothelial growth factor (VEGF) and its receptor VEGFR2 (20, 21, 22). Thus, loss of barrier function in quiescent EC in combination with defective angiogenic capacity in proliferative EC would enhance the progression and severity of ALI. Therefore, identifying a mechanism that controls the functions of EC both in their quiescent and proliferative phases may lead to the development of a more effective therapeutic strategy to control ALI.

Mechanistic (formerly mammalian) target of rapamycin (MTOR) is a kinase with essential roles in protein synthesis, cell growth, metabolism, and cytoskeletal organization (23, 24, 25, 26). MTOR signals in two distinct complexes, MTOR complex 1 (MTORC1) and MTOR complex 2 (MTORC2) to exert these functions. MTORC1, characterized by the presence of rapamycin-sensitive regulatory associated protein of MTOR, is a nutrient-responsive signaling complex regulating cell growth and metabolism. MTORC1 controls these responses in major part *via* phosphorylation of at least two regulators of protein synthesis: ribosomal protein S6 kinase and an inhibitor of translation initiation, eukaryotic initiation factor 4E-binding protein 1 (27, 28, 29, 30). MTORC2, defined by the presence of rapamycin-insensitive companion of MTOR, activates Akt and PKCα pathways to stimulate cell proliferation, survival, and actin reorganization (29, 31, 32). Dysregulated MTOR signaling has been implicated in the progression of several diseases including chronic obstructive pulmonary disease, pulmonary arterial hypertension, idiopathic pulmonary fibrosis, heart failure, and cancer (33, 34, 35, 36, 37, 38, 39, 40, 41, 42). We have previously shown a role of MTOR in suppressing inflammation in the activated endothelium *in vitro* (43, 44). Similarly, Chen *et al.* recently reported a role of endothelial MTOR in protecting against lung injury in mice with endotoxemia (45). However, it is unclear if MTOR plays a role in preserving the integrity of the quiescent endothelium and proliferative/migratory capacity of EC after injury, and whether loss of endothelial MTOR is a vital component of lung vascular injury. Here, we show that MTOR is downregulated in activated EC and silencing *MTOR* in EC alters cell morphology, disrupts barrier function, and impairs cell proliferation and migration. Similarly, MTOR level is markedly reduced in injured lungs and depleting or deleting endothelial *MTOR* increases lung vascular permeability and edema even in the absence of ALI-causing conditions. Consistent with the reduced barrier function and proliferative/migratory capacity, VE-cadherin and VEGFR2 levels are decreased in *MTOR* knockdown EC as well as in the lungs of mice with EC-specific MTOR deficiency. Importantly, expressing MTOR in lung endothelium, either prophylactically or therapeutically, mitigates inflammatory lung injury and improves lung function and survival in mice. Together, these data establish the essential role played by endothelial MTOR in maintaining lung vascular integrity and identify it as a potential therapeutic target to control ALI and improve survival in mice.

## Results

### MTOR is necessary to maintain EC barrier integrity

**Figure 1:**
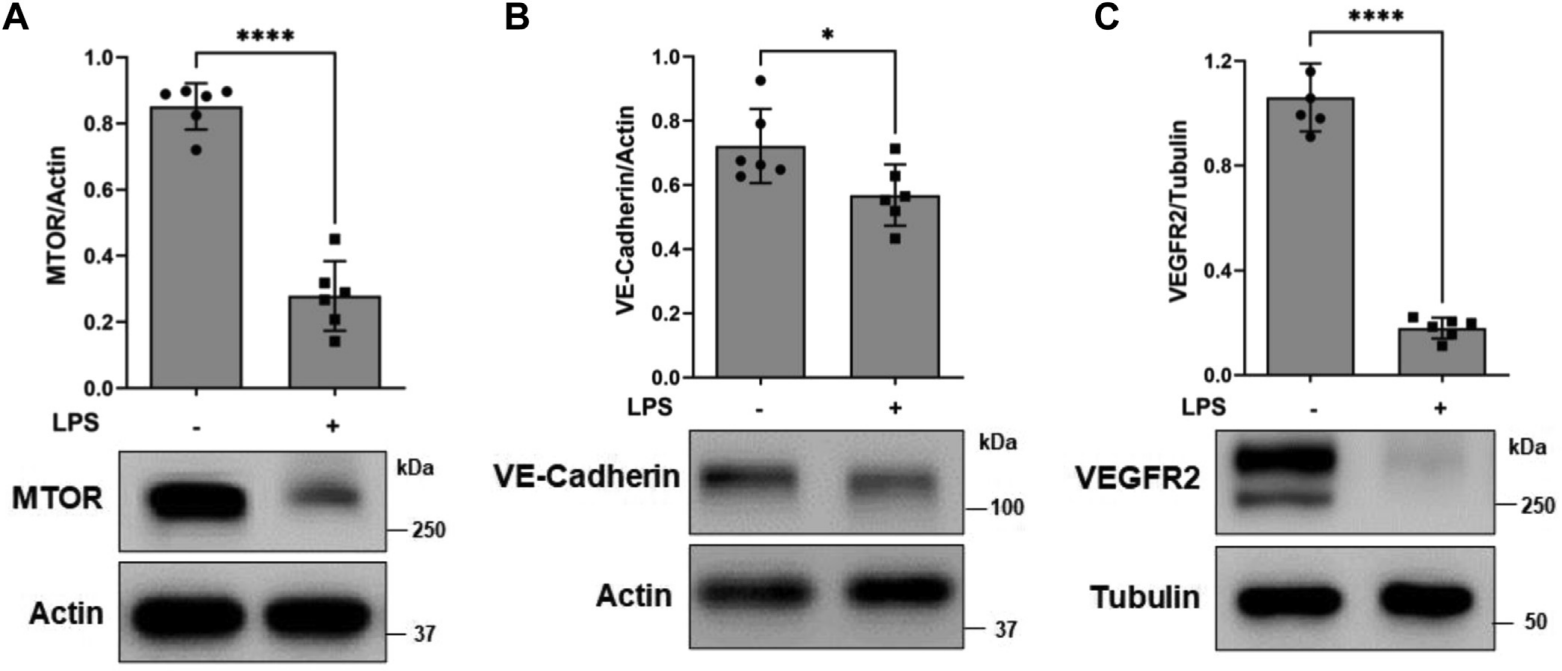
**LPS decreases MTOR, VE-cadherin, and VEGFR2 levels in EC.** HPAECs were challenged with LPS (5 μg/ml) for 6 h. Cells were lysed and analyzed by Western blot to determine the levels of (*A*) MTOR, (*B*) VE-cadherin, or (*C*) VEGFR2. Actin or Tubulin levels were used to monitor loading. Bars indicate mean ± SD (n = 6 per condition). ∗*p* < 0.05 and ∗∗∗∗*p* < 0.0001 by Student’s *t* test. EC, endothelial cell; LPS, lipopolysaccharide; MTOR, mechanistic target of rapamycin; VEGFR, vascular endothelial growth factor receptor; VE, vascular endothelial.

**Figure 2:**
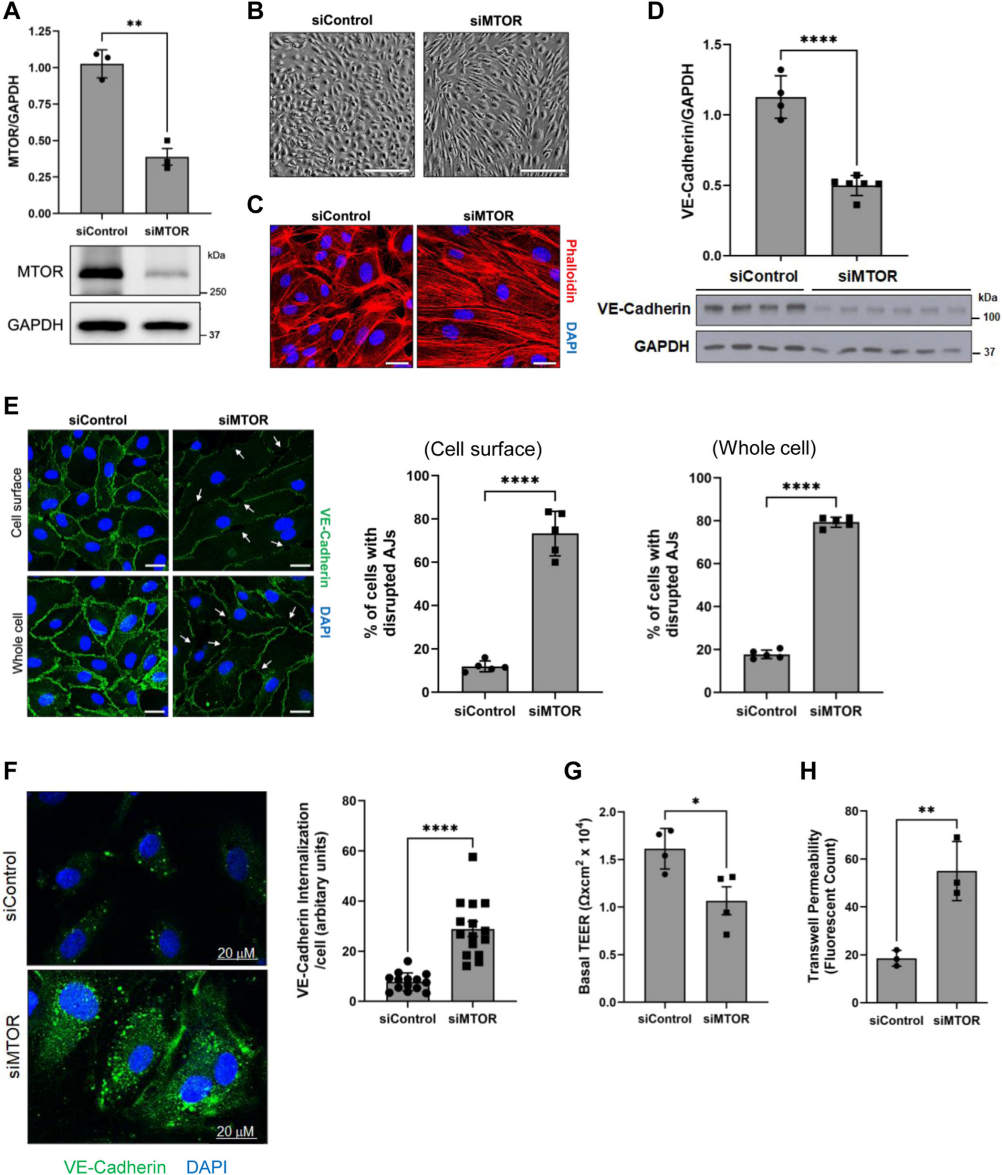
**MTOR maintains cell morphology and barrier integrity in EC.***A*, HPAECs were transfected with Control or MTOR siRNA (siControl or siMTOR) for 48 h. Cells were lysed, and Western blot analysis was performed to determine MTOR and GAPDH levels. Bars indicate the effect of siRNA on MTOR level normalized to GAPDH. Data are mean ± SD (n = 3 for each condition). *B*, siControl and siMTOR cells were grown to confluency and cell morphology was assessed. The scale bar represents 200 μm. *C*, HPAECs were plated on glass coverslips and transfected with siControl or siMTOR for 48 h. Cells were fixed and stained with phalloidin to mark actin stress fibers (*red*). *D*, siControl and siMTOR cell lysates were analyzed by Western blot to determine the levels of VE-cadherin and GAPDH. Bars indicate the effect of MTOR siRNA on VE-cadherin level normalized to GAPDH. Data are mean ± SD (n = 4–6) for each condition. *E*, HPAECs were plated on glass coverslips and transfected with siControl or siMTOR for 48 h. Cells were fixed and stained with anti-VE-cadherin antibody (*green*) to mark cell surface (*upper panels*) or whole cell (*lower panels*) VE-cadherin. 4',6-diamidino-2-phenylindole (*blue*) was used to stain nuclei. The scale bar represents 25 μm, and *white arrows* indicate regions with disrupted cell surface VE-cadherin staining. The percent of cells with disrupted AJs in cell surface or whole cell staining was counted as described in the “Experimental procedures” section. Error bars represent mean ± SD (n = 5 with 28–64 cells counted in every field for each condition). *F*, HPAECs grown on glass coverslips were transfected with siControl or siMTOR siRNA for 48 h and then labeled with anti-VE-cadherin antibody in the presence of 100 μM chloroquine for 4 h at 37 °C. Internalization of VE-cadherin (*green puncta*) was examined by confocal microscopy and normalized to the total number of cells in each field. The scale bar represents 20 μm. Bars indicate mean ± SD (n = 14–15 fields from three coverslips per condition). *G*, HPAECs were transfected with siControl or siMTOR for 48 h, transferred to gold electrode arrays, and grown to confluency. Basal transendothelial electrical resistance (TEER) was measured by Electric Cell-substrate Impedance Sensing (ECIS). Bars indicate mean ± SD (n = 4) for each condition. *H*, HPAECs transfected with siControl or siMTOR were seeded on transwell inserts and allowed to grow to confluency. FITC-labeled Dextran was added to the culture media on top of the inserts and permeabilized Dextran concentrations were measured in the receiver tray (n = 3/condition). ∗*p* < 0.05, ∗∗*p* < 0.01, and ∗∗∗∗*p* < 0.0001 by Student’s *t* test. AJ, adherens junction; EC, endothelial cell; HPAEC, human pulmonary artery endothelial cell; MTOR, mechanistic target of rapamycin; VE, vascular endothelial.

To address the role of MTOR in preserving EC function, we first determined if MTOR level is altered in EC stimulated with lipopolysaccharide (LPS), a robust activator of EC dysfunction. LPS induced a substantial decrease in MTOR level (Fig. 1*A*). Similarly, the levels of VE-cadherin and VEGFR2, important regulators of EC barrier function and proliferative/migratory capacity were also decreased (Fig. 1, *B* and *C*). These results led us to determine if loss of MTOR is an important determinant of EC dysfunction. To this end, we first determined the effect of silencing *MTOR* using siRNA on EC barrier integrity. Depletion of MTOR by this approach (Fig. 2*A*) elicited significant morphological changes, characterized by elongated shape with pointed ends (Fig. 2*B*). Staining of actin cytoskeleton and VE-cadherin further confirmed the profound cell shape change caused by *MTOR* knockdown (Fig. 2, *C* and *E*). MTOR-depleted cells displayed prominent actin stress fibers that seem to extend from one end of the cell to the other (Fig. 2*C*). Moreover, these cells also showed a decrease in both total and cell surface VE-cadherin (Fig. 2, *D* and *E*). The cell surface VE-cadherin was also marked by areas of discontinuity in *MTOR* knockdown cells (Fig. 2*E*; white arrows). The loss of cell surface VE-cadherin was primarily a result of increased endocytosis of VE-cadherin in MTOR-depleted cells (Fig. 2*F*). Together, these observations suggest a role of MTOR in preserving EC barrier integrity in part by preventing the endocytosis of cell surface VE-cadherin. We tested this possibility by monitoring (i) transendothelial electrical resistance (TEER) of EC grown on gold microelectrodes (a highly sensitive method for real-time measurement of barrier integrity) and (ii) FITC-Dextran flux across the EC barrier using the transwell assay, a direct measure of EC permeability. A marked decrease in TEER was noted in MTOR-depleted cells (Fig. 2*G*). Consistent with this, FITC-labeled Dextran flux was greatly increased in the absence of MTOR (Fig. 2*H*). These data indicate an important role for MTOR in maintaining EC barrier function by preserving AJ integrity.

### MTOR is necessary to maintain EC proliferative and migratory capacity

**Figure 3:**
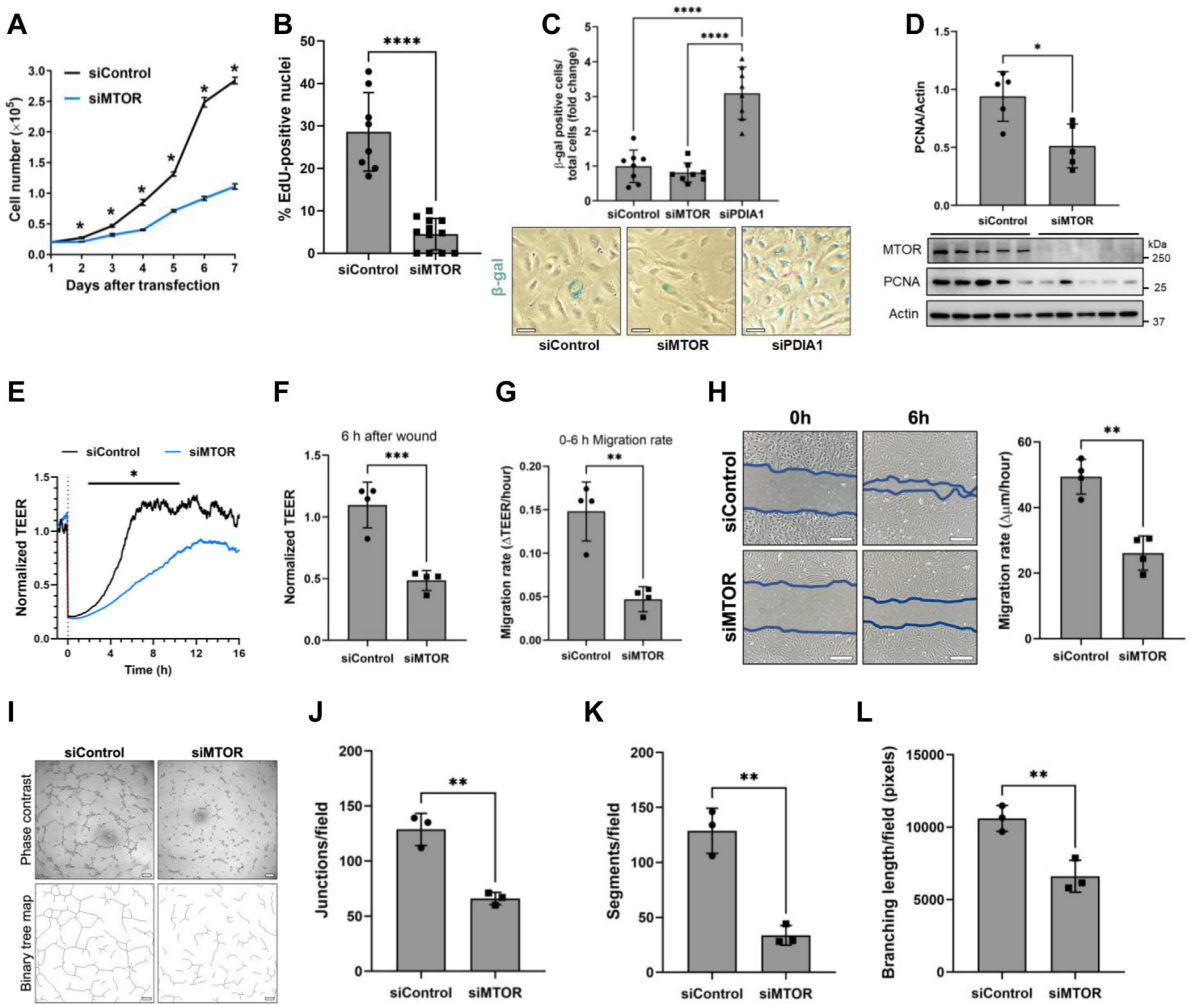
**MTOR is essential for EC proliferation and migration.***A*, HPAECs were transfected with siControl or siMTOR for 24 h before replating equal cell numbers at subconfluent density. Cells were trypsinized and counted daily to assess cell proliferation. Data indicate mean ± SD (n = 3 wells per time point). *B*, HPAECs were transfected for 24 h with siControl or siMTOR, and equal cell numbers were replated on glass coverslips at subconfluent density. After 48 h transfection, cells were incubated with EdU for 6 h and detected according to Click-iT EdU assay protocol. The number of EdU-positive nuclei was normalized to Hoescht-positive nuclei in each field. Bars indicate mean ± SD (n = 8–12 fields from three coverslips per condition). *C*, HPAECs, transfected with siControl or siMTOR (or siPDIA1 as a positive control), were allowed to grow for 144 h before fixation and detection of β-galactosidase (β-gal, *blue*) activity. The number of β-gal positive cells was normalized to the total number of cells in each field and fold change compared to siControl was determined. Bars indicate mean ± SD (n = 8 fields from four wells per condition), and the scale bar represents 50 μm. *D*, cell lysates from siControl and siMTOR HPAECs were analyzed by Western blot to determine MTOR, PCNA, and actin levels. Bars indicate the effect of siRNA on PCNA level normalized to actin. Data are mean ± SD (n = 4 for each condition). *E*–*G*, siControl and siMTOR HPAECs were transfected for 48 h, transferred to gold electrode arrays, and allowed to grow to confluency. *E*, a wound was induced (*red line*, 0 h) and transendothelial electrical resistance (TEER) was measured over time by Electric Cell-substrate Impedance Sensing (ECIS). TEER readings were normalized to prewound measurements. *F*, normalized TEER readings 6 h after wounding and (*G*) the cell migration rate from 0 to 6 h. Bars indicate mean ± SD (n = 4 wells per condition). *H*, confluent monolayers of siControl and siMTOR HPAECs were scratched and washed with fresh media. Cells were photographed immediately after washing and after 6 h incubation. Wound width was calculated at four regions per well at 0 h and 6 h and averaged to calculate the cell migration rate. Bars indicate mean ± SD (n = 4 wells per condition), and the scale bar represents 200 μm. *I*–*L*, siControl and siMTOR HPAECs were plated at subconfluent density in Matrigel matrix and incubated for 6 h and then photographed. *I*, binary tree maps were created to visualize tube formation, and network structure in each field was quantitated to determine the number of (*J*) junctions or branching points, (*K*) segments or lines connecting two junctions, and (*L*) the branching length was calculated as the sum of the length of the interconnected tube network. Bars indicate mean ± SD (n = 3 wells per condition), and the scale bar represents 200 μm. ∗*p* < 0.05, ∗∗*p* < 0.01, ∗∗∗*p* < 0.001, and ∗∗∗∗*p* < 0.0001 by Student’s *t* test. PCNA, proliferating cell nuclear antigen; HPAEC, human pulmonary artery endothelial cell; MTOR, mechanistic target of rapamycin.

EC have unique angiogenic properties that allow them to proliferate and migrate to form new blood vessels or restore the barrier function after injury. Hence, we examined if *MTOR* knockdown influences the proliferative and migratory capacity of these cells (18, 19). MTOR-depleted ECs were severely impaired in their ability to proliferate (Fig. 3, *A* and *B*) but exhibited no decrease in viability (Fig. S1). We also determined if loss of MTOR promotes a senescence-like phenotype in EC to dampen their proliferative capacity. MTOR-depleted cells showed no evidence of senescence as determined by β-galactosidase activity, whereas knockdown of protein disulfide isomerase 1 (used as a positive control) resulted in senescence of EC (Fig. 3*C*) as reported (46). We next asked if the expression of proliferating cell nuclear antigen (PCNA), a nuclear nonhistone protein that plays an essential role in cell cycle regulation and is used as a marker for cells in early G1/S phase, is also altered in MTOR-depleted cells (47). PCNA was clearly decreased in these cells (Fig. 3*D*), pointing to a block in the entry into S phase following *MTOR* knockdown. These data support a role of MTOR in maintaining EC proliferative capacity. We next evaluated the effect of *MTOR* knockdown on the migratory capacity of EC. This response was strongly inhibited in MTOR-depleted EC as evidenced by slow recovery of TEER after wounding (Fig. 3, *E*–*G*). These results were further confirmed by reduced rate of wound closure in a scratch assay (Fig. 3*H*). Notably, both TEER and scratch assays revealed a similar rate of EC migration (Fig. 3, *G* and *H*). Consistent with the deficiencies in proliferation and migration, MTOR-depleted EC exhibited impaired tube formation capacity (Fig. 3, *I–L*).

**Figure 4:**
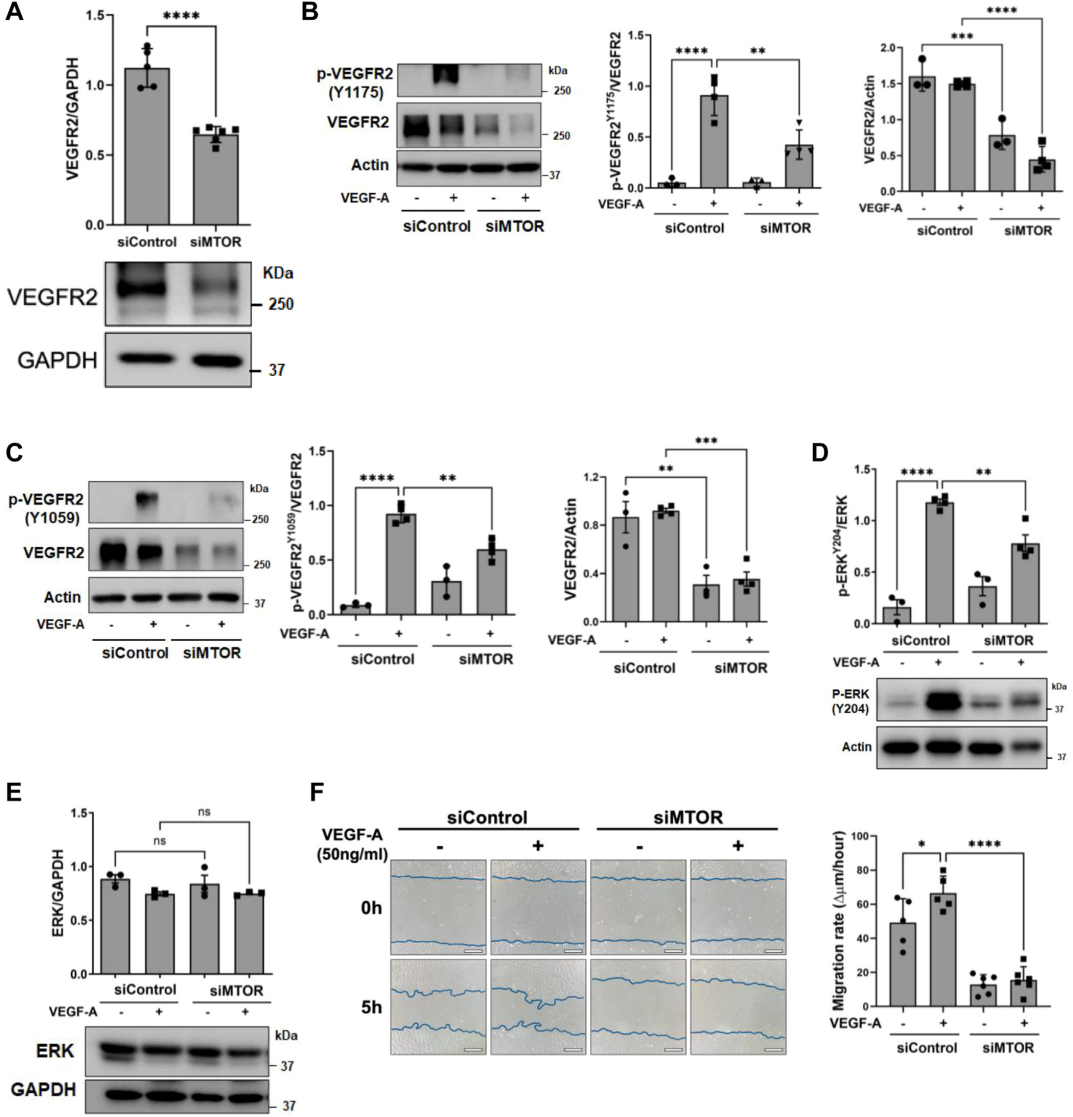
**VEGF response is abolished in MTOR-depleted EC.***A*, cell lysates from HPAEC transfected with siControl or siMTOR for 48 h were analyzed by Western blot to determine VEGFR2 and GAPDH protein levels. Bars indicate mean ± SD (n = 5–6 per condition). *B*–*G*, HPAECs were transfected with siControl and siMTOR for 48 h and were serum-starved (0.5% fetal bovine serum) for 12 h and then treated with VEGF-A (50 ng/ml) for 5 min. Cell lysates were analyzed by Western blot for (*B*) phospho-VEGFR2^Y1175^, total VEGFR2 and actin levels, (*C*) phospho-VEGFR2^Y1059^, total VEGFR2 and actin levels, (*D*) phospho-ERK1/2^Y204^, and total ERK (*E*) total ERK and actin. Bars indicate mean ± SD (n = 3–4 per condition). *F*, confluent monolayers of siControl and siMTOR HPAECs were scratched and washed with media, then incubated with fresh media containing VEGF-A (50 ng/ml) where indicated. Cells were photographed immediately after washing and after 5 h incubation. Wound width was calculated at four regions per well at 0 h and 5 h and averaged to calculate the cell migration rate. Bars indicate mean ± SD (n = 5–6 wells for each condition), and the scale bar represents 200 μm. ∗*p* < 0.05, ∗∗*p* < 0.01, ∗∗∗*p* < 0.001, and ∗∗∗∗*p* < 0.0001 by one-way ANOVA. EC, endothelial cell; HPAEC, human pulmonary artery endothelial cell; MTOR, mechanistic target of rapamycin; VE, vascular endothelial; VEGF, vascular endothelial growth factor; VEGFR, vascular endothelial growth factor receptor.

Given the established role of VEGF in controlling the migratory capacity of EC (20, 21, 22) and the loss of MTOR and VEGFR2 in LPS-stimulated EC (Fig. 1, *A* and *C*), we assessed if *MTOR* knockdown affects the expression of VEGFR2. VEGFR2 level was markedly reduced in MTOR-depleted EC (Fig. 4*A*). Moreover, MTOR-depleted cells showed reduced phosphorylation (Y1175 and Y1059) of VEGFR2 when stimulated with VEGF-A (Fig. 4, *B* and *C*). Thus, in addition to the decreased level of VEGFR2, the activation capacity of the remaining VEGFR2 was also inhibited by *MTOR* knockdown, signifying a role of MTOR both in maintaining the expression and activation of the receptor. To corroborate the reduced VEGFR2 activation in MTOR-depleted EC, we determined the activation status of the VEGF-responsive kinase ERK1/2. As expected, the level of phosphorylated but not total ERK1/2 was significantly decreased in EC after MTOR depletion (Fig. 4, *D* and *E*). We also determined the status of Akt and eNOS level/phosphorylation in *MTOR* knockdown cells. However, given the loss of VEGFR2 level and VEGF signaling in *MTOR* knockdown cells (Fig. 4, *B–E*), we performed the experiments only in the absence of VEGF stimulation. We found that Akt phosphorylation was slightly increased, but Akt level remained unaltered in *MTOR* knockdown cells compared to control cells (Fig. S2*A*). These observations are consistent with an earlier report by Wang *et al.* (48). In contrast with the above study (48) which showed a decrease in eNOS level but not its phosphorylation, both eNOS level and phosphorylation remained unchanged in MTOR-depleted human pulmonary artery endothelial cell (HPAEC) (Fig. S2*B*). The exact reason for this discrepancy is not clear. One possible explanation could be the use of HPAEC in our study *versus* human aortic endothelial cells used by Wang *et al.* (48).

We next determined whether the loss of VEGFR2 and its downstream signaling renders MTOR-depleted cells refractory to cell migration induced by VEGF. VEGF-A increased scratch closure in control cells, as expected. However, it failed to do so in MTOR-depleted cells (Fig. 4*F*). Collectively, these data indicate that MTOR preserves EC migratory and tube formation capacity by maintaining VEGFR2 signaling.

### MTOR preserves VE-cadherin and VEGFR2 levels by suppressing autophagy

The loss of VE-cadherin and VEGFR2 prompted us to examine the possibility that MTOR-depleted EC are undergoing endothelial-to-mesenchymal transition (EndMT). Our results show that the loss of these endothelial proteins (Figs. 2*D* and 4*A*) is not accompanied by an increase in mesenchymal markers in MTOR-depleted EC (Fig. S3). In fact, mesenchymal markers are either reduced or remain unaffected in these cells (Fig. S3). These data argue against a connection between MTOR depletion and EndMT under the condition used in this study.

**Figure 5:**
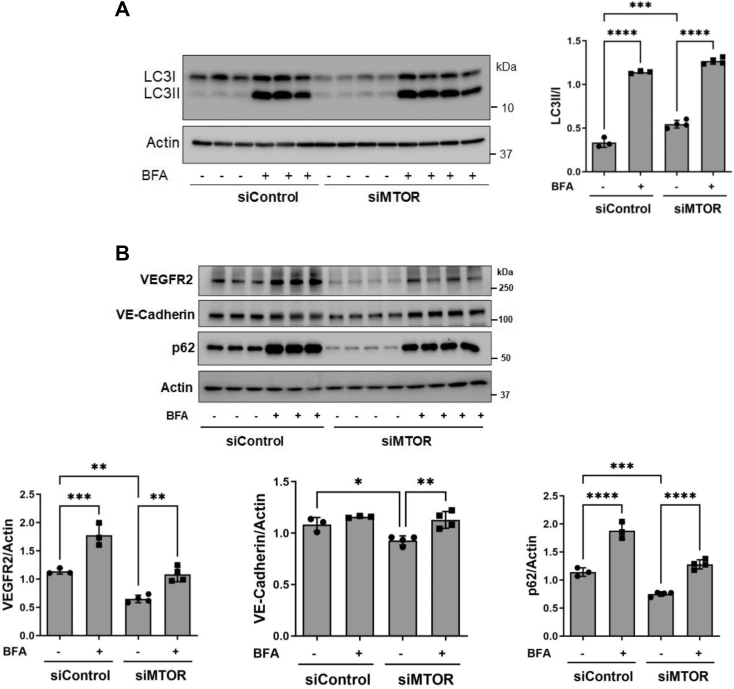
**Bafilomycin A1 prevents the loss of VEGFR2 and VE-cadherin in MTOR-depleted EC.** HPAECs were transfected with siControl or siMTOR for 24 h and then treated with BFA (10 nM). Twenty-four hours post-BFA treatment, cell lysates were immunoblotted for (*A*) LC3II and LC3I and (*B*) VEGFR2, VE-cadherin, and p62 levels. Actin was used to monitor protein loading. Bars indicate mean ± SD (n = 3–4/condition). ∗*p* < 0.05, ∗∗*p* < 0.01, ∗∗∗*p* < 0.001, and ∗∗∗∗*p* < 0.0001 by one-way ANOVA. EC, endothelial cell; MTOR, mechanistic target of rapamycin; VE, vascular endothelial; VEGFR, vascular endothelial growth factor receptor.

Because inhibition of autophagy increases cell surface VE-cadherin and EC barrier integrity (49), we determined if autophagy plays a role in mediating the loss of VE-cadherin and VEGFR2 induced by *MTOR* silencing. As expected, *MTOR* knockdown cells exhibited increased autophagy flux as evidenced by increased LC3II/LC3I ratio and decreased p62 levels (Fig. 5, *A* and *B*). Inhibition of autophagy by bafilomycin A1 prevented the loss of VE-cadherin and VEGFR2 in MTOR-depleted cells (Fig. 5*B*). These results indicate that MTOR preserves VE-cadherin and VEGFR2 levels in part by its ability to inhibit autophagy.

### Endothelial MTOR protects against lung vascular injury

**Figure 6:**
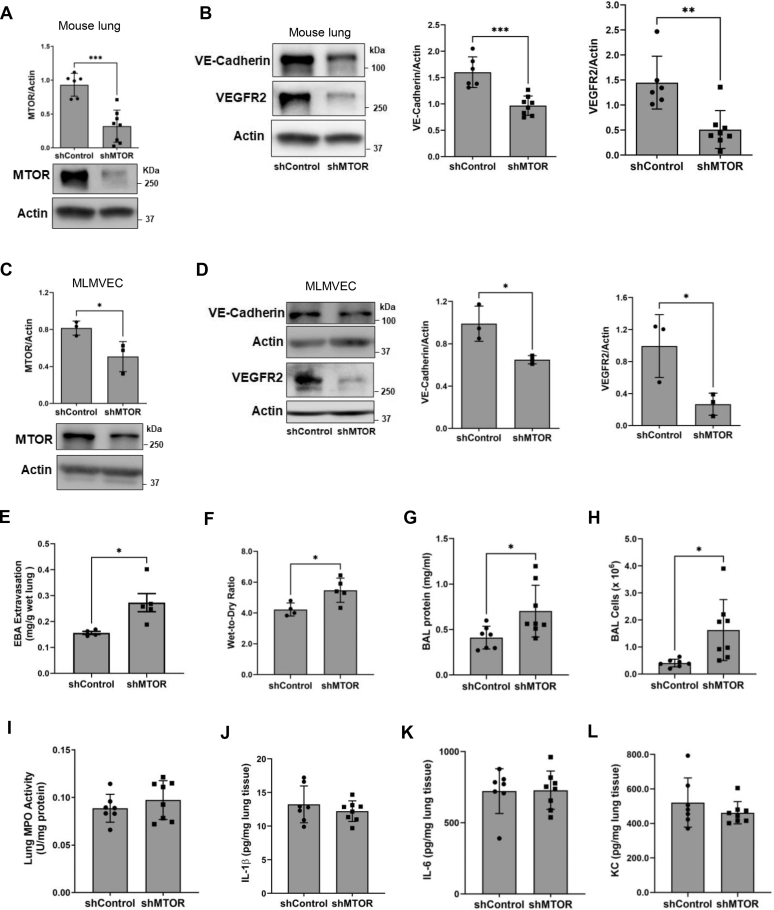
**EC-targeted MTOR depletion causes lung vascular leak in mice.** MTOR was selectively depleted in the lung EC of WT B6 mice *via* cationic liposome-mediated gene transfer of MTOR shRNA-expressing plasmids (shMTOR). Mice were euthanized 3 days after gene transfer, and BAL fluid and lung tissue were extracted. Mice given plasmid with control shRNA (shControl) were used as controls. *A* and *B*, lungs from shControl and shMTOR mice were homogenized and protein lysates were analyzed by Western blot for (*A*) MTOR and (*B*) VE-cadherin and VEGFR2 levels. Actin levels were used to monitor loading. Bars represent mean ± SD (n = 6–8 per condition). *C* and *D*, lung microvascular endothelial cells (LMVECs) from shControl and shMTOR mice were isolated as described in Experimental procedures. Cell lysates were analyzed for (*C*) MTOR or (*D*) VE-cadherin and VEGFR2 levels. Bars represent mean ± SD (n = 3/condition). *E* and *F*, Evans blue albumin (EBA) was delivered by retro-orbital vein injection to shControl and shMTOR mice 1 h before euthanasia. EBA extravasation (*E*) and lung wet-to-dry weight ratio (*F*) were measured to assess lung vascular permeability and lung edema, respectively. Bars represent mean ± SD (n = 4–5 mice/condition). *G* and *H*, BAL fluid collected from the lungs of shControl and shMTOR mice was analyzed for (*G*) protein content and (*H*) the number of infiltrated cells. *I*–*L*, lung homogenates from shControl and shMTOR mice were analyzed for (*I*) MPO activity, (*J*) interleukin-1 beta (IL-1β), (*K*) IL-6, and (*L*) keratinocyte chemoattractant (KC) levels. Bars represent mean ± SD (n = 7–8 mice per condition). ∗*p* < 0.05, ∗∗*p* < 0.01, and ∗∗∗*p* < 0.001 by Student’s *t* test. BAL, bronchoalveolar lavage; EC, endothelial cell; MPO, myeloperoxidase; MTOR, mechanistic target of rapamycin; VE, vascular endothelial; VEGFR, vascular endothelial growth factor receptor.

The finding that MTOR regulates essential EC functions led us to investigate the role of endothelial MTOR *in vivo*. We first determined if the *in vitro* effect of MTOR depletion on EC barrier disruption can be recapitulated in the lung. We used cationic liposome-mediated gene transfer to selectively target the lung endothelium for knockdown of *MTOR* *via* transfer of MTOR shRNA expressing plasmid (shMTOR) in WT mice. Western blot analysis of lung homogenates showed that depletion of MTOR by this approach (Fig. 6*A*) caused a marked decrease in VE-cadherin and VEGFR2 levels (Fig. 6*B*), consistent with our data from cultured EC (Figs. 2*D* and 4*A*). Similarly, lung microvascular endothelial cells (LMVECs) isolated from these mice also showed a decrease in MTOR, VE-cadherin, and VEGFR2 levels (Fig. 6, *C* and *D*), confirming the knockdown of *MTOR* and loss of VE-cadherin and VEGFR2 in the lung endothelium with MTOR depletion. These cells also did not show an increase in mesenchymal markers (Fig. S4), consistent with the data in MTOR-depleted HPAEC (Fig. S3).

We next examined the effect of MTOR loss in the lung endothelium on lung vascular injury. Mice with lung EC-MTOR depletion showed an increase in pulmonary extravasation of Evans blue albumin and leakage of serum albumin into the bronchoalveolar lavage (BAL) fluid, markers of vascular permeability, and lung wet-to-dry weight ratio, an important index of pulmonary edema (Fig. 6, *E*–*G*), and immune cell recruitment (Fig. 6*H*) compared to mice transduced with control shRNA plasmid (shControl). Further analysis of these lungs showed that despite the increase in total BAL cells, other markers of inflammation (increase in myeloperoxidase [MPO] activity and proinflammatory mediators) remained unchanged in shMTOR-transduced mice (Fig. 6, *I*–*L*). These data indicate that knockdown of EC-*MTOR* is sufficient to cause lung vascular leakage without inducing inflammation and point to the possibility that increased BAL cells could be a result of disrupted EC barrier in shMTOR lungs. These data show that loss of EC-MTOR is an important component of lung vascular injury.

**Figure 7:**
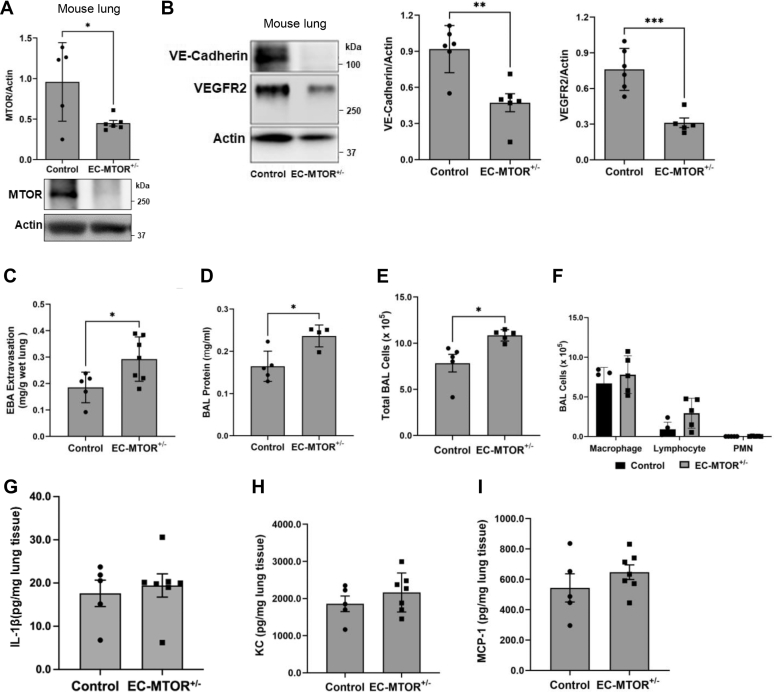
**EC-specific *Mtor* deletion causes lung vascular injury in mice.***Mtor*^*fl/fl*^ mice were crossed with heterozygous *VE-cadherin*^*Cre+*^ mice which express Cre recombinase under control of VE-cadherin promoter. The resulting EC-specific heterozygous MTOR KO mice (*VE-cadherin*^*C*^^*re*^^*+*^/*Mtor*^*+/fl*^ [EC-*Mtor*^*+/−*^] were analyzed for markers of lung vascular injury and inflammation. Mice with intact EC MTOR (*Mtor*^*fl/fl*^ [Control]) mice were used as controls. *A* and *B*, lungs from EC-*Mtor*^*+/−*^ and control mice were homogenized, and protein lysates were analyzed by Western blot for (*A*) MTOR and (*B*) VE-cadherin and VEGFR2 levels. Actin levels were used to monitor loading. *C*, Evans blue albumin (EBA) extravasation was measured to determine lung vascular permeability. *D* and *E*, BAL fluid collected from control and EC-*Mtor*^*+/−*^ mice was analyzed for (*D*) protein content and (*E*) the number of infiltrated cells. *F*, differential staining of BAL cells was performed to determine the number of macrophages, lymphocytes, and polymorphonuclear leukocytes (PMN) in the lungs of control and EC-*Mtor*^*+/−*^ mice. *G–I*, lung homogenates from control and EC-*Mtor*^*+/−*^ mice were analyzed for (*G*) IL-1β, (*H*) KC, and (*I*) monocyte chemoattractant protein-1 (MCP-1) levels. Bars represent mean ± SD (n = 4–7 mice [male and female] per condition). ∗*p* < 0.05, ∗∗*p* < 0.01, and ∗∗∗*p* < 0.001 by Student’s *t* test. BAL, bronchoalveolar lavage; EC, endothelial cell; IL, interleukin; KC, keratinocyte chemoattractant; MTOR, mechanistic target of rapamycin; VE, vascular endothelial; VEGFR, vascular endothelial growth factor receptor.

Transgenic mice with EC-specific deletion of *MTOR* were used to further validate these data. *Mto*r^*fl/fl*^ mice were bred with *VE-cadherin*^C^^*re*^ mice to generate endothelial-specific heterozygous MTOR KO (*Mtor*^*fl/+*^*/VE-cadherin*^*Cre*^^*+*^ [EC-*Mtor*^*+/−*^]) mice (Fig. S5), and the levels of MTOR, VE-cadherin, and VEGFR2 in the lungs of these mice were evaluated by immunoblotting. The level of MTOR was markedly decreased in the lungs of EC-*Mtor*^*+/−*^ mice (Fig. 7*A*). A similar decrease in VE-cadherin and VEGFR2 level was also observed in these lungs (Fig. 7*B*). EC-*Mtor*^*+/*−^ lungs also exhibited increased lung vascular permeability and immune cell infiltration (Fig. 7, *C*–*E*). Intriguingly, differential staining of BAL cells revealed that majority of infiltrated cells in EC-*Mtor*^*+/−*^ lungs were lymphocytes, and polymorphonuclear leukocytes (PMN) comprised only a minute fraction (Fig. 7*F*). Similarly, no increase in the levels of proinflammatory mediators was noted in the lungs from these mice (Fig. 7, *G*–*I*). These data further verify that loss of EC-MTOR is a critical determinant of lung vascular injury.

**Figure 8:**
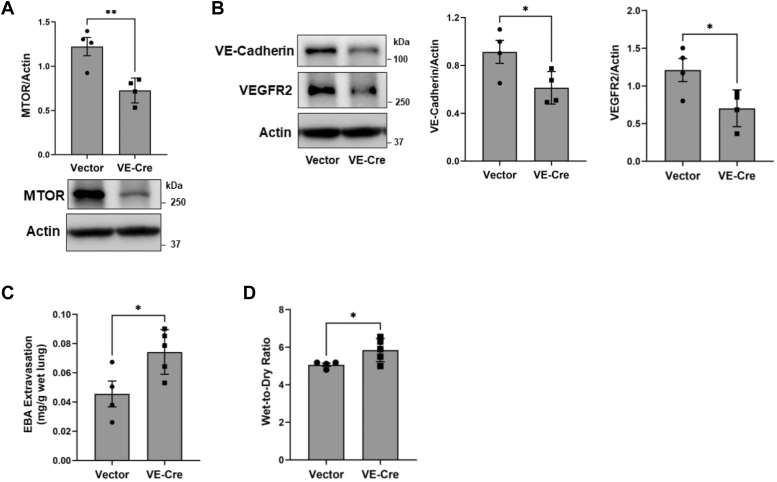
**Deletion of *Mtor* in the lung endothelium *via* pVECad-Cre reduces lung vascular injury in mice.***Mtor*^*fl/fl*^ mice were transduced with pVECad-Cre (pVE-Cre) *via* cationic liposomes. After 10 days, mice were injected with Evans blue albumin (EBA) 1 h before euthanasia and lungs were analyzed for (*A*) MTOR level and (*B*) VE-Cadherin and VEGFR2 levels by Western blot, (*C*) EBA extravasation, and (*D*) lung wet-to-dry weight ratio. Bars represent mean ± SD (n = 4–5 mice per condition). ∗*p* < 0.05, ∗∗*p* < 0.01 by Student’s *t* test. MTOR, mechanistic target of rapamycin; VE, vascular endothelial; VEGFR, vascular endothelial growth factor receptor.

We also used our recently described gene transfer–based approach (50) to delete the *MTOR* gene in the lung endothelium and assess if it reproduces the effect of *MTOR* deletion by the above transgenic approach on lung vascular permeability and edema. Briefly, a construct (pVECad-CRE) containing Cre recombinase complementary DNA (cDNA) under the control of EC-specific VE-cadherin promoter was delivered in *Mtor*^*fl/fl*^ mice *via* cationic liposomes. Lungs from these mice showed decreased MTOR, VE-cadherin, and VEGFR2 levels (Fig. 8, *A* and *B*) but increased lung vascular permeability and water content compared to control mice (*Mtor*^*fl/fl*^ transduced with empty vector [pRK5] (Fig. 8, *C* and *D*). Together, these data show that the MTOR deficiency in the lung endothelium is sufficient to render the lung leaky and establish the essential role played by MTOR in maintaining lung vascular integrity.

### Prophylactic MTOR expression in the lung endothelium protects against ALI

**Figure 9:**
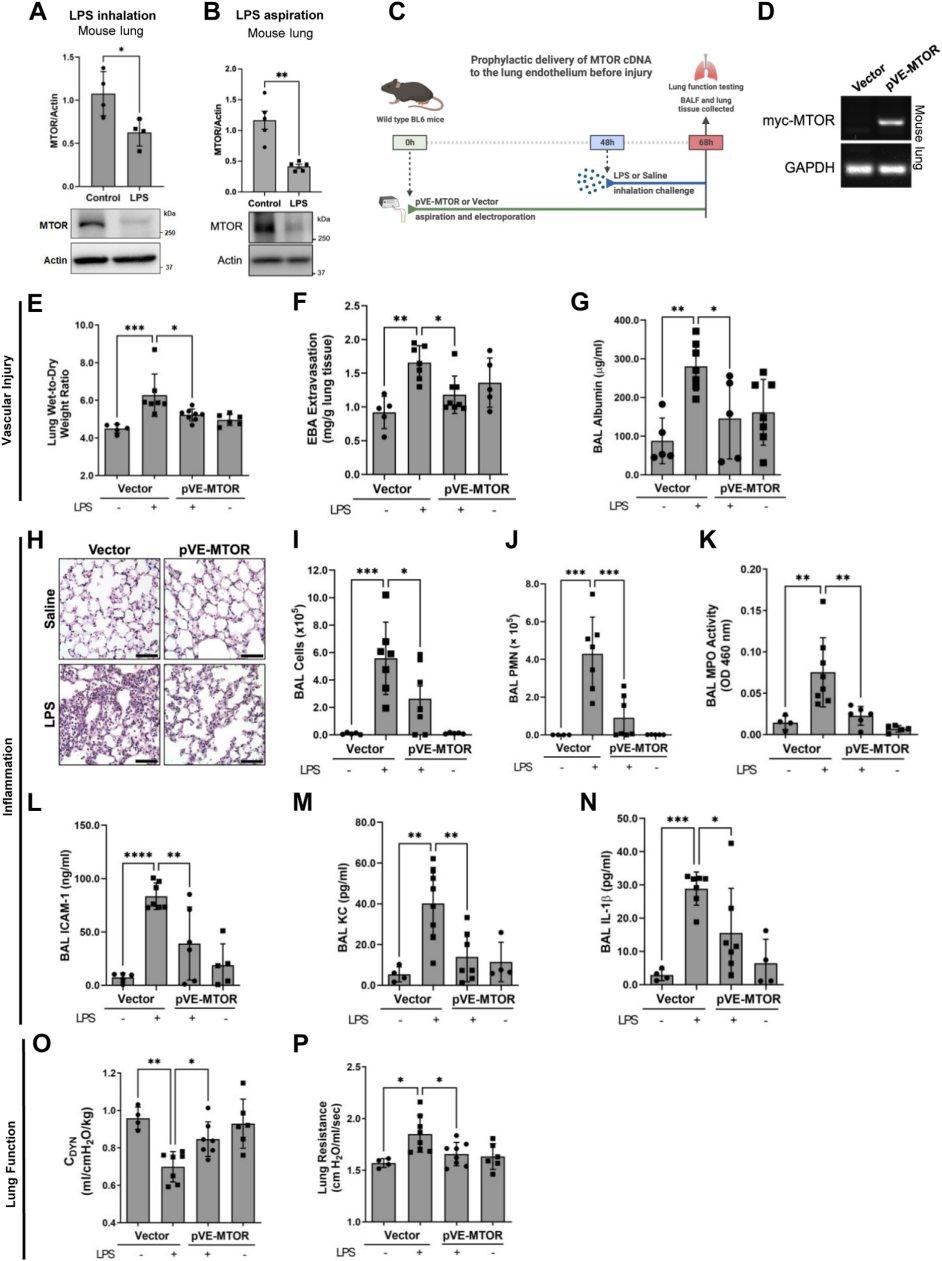
**MTOR expression in the lung endothelium protects against LPS-induced lung injury and dysfunction.***A* and *B*, MTOR is reduced in mice with ALI. WT B6 mice were challenged with (*A*) LPS inhalation (1 mg/ml in 6 ml PBS or saline) for 18 to 20 h or (*B*) LPS aspiration (5 mg/kg) for 72 h as described in Experimental Procedures, and lungs were analyzed by Western blot for MTOR and actin levels. Bars represent mean ± SD (n = 4–5 mice per condition). ∗*p* < 0.05, ∗∗*p* < 0.01 by Student’s *t* test. *C–P*, prophylactic MTOR expression in lung EC reduces inflammatory injury and improves lung function. *C*, MTOR was expressed in the lung endothelium of WT B6 mice *via* electroporation-mediated gene transfer of pVE-MTOR in which myc-tagged MTOR complementary DNA plasmid is driven by EC-specific VE-cadherin (VE) promoter. Mice given empty plasmid (vector) were used as controls. Forty-eight hours after gene transfer, mice were aerosolized with 6 ml of saline or saline containing *Escherichia coli* LPS (1 mg/ml) for 1 h. At 18 to 20 h after LPS challenge, BAL fluid and lung tissue were collected for various analyses. *D*, RNA was extracted from pVE-MTOR or vector mouse lungs and analyzed by RT-PCR to confirm the expression of myc-MTOR from the delivered pVE-MTOR plasmid. GAPDH mRNA expression was used to monitor the loading. *E–G*, lung wet-to-dry weight ratio (*E*), EBA extravasation (*F*), and BAL albumin (*G*) were determined as markers of lung vascular injury. *H*, lung histology and inflammatory cell recruitment was determined by H&E staining. *I–K*, lung polymorphonuclear leukocytes (PMN) infiltration was assessed by measuring (*I*) BAL cells, (*J*) BAL PMN, and (*K*) BAL MPO activity. *L–N*, BAL fluid was analyzed by ELISA to assess the levels of the proinflammatory mediators (*L*) ICAM-1, (*M*) KC, and (*N*) IL-1β. *O* and *P*, lung function was assessed by measuring the (*O*) dynamic compliance and (*P*) resistance of lungs from mice transduced with pVE-MTOR or vector followed by LPS or saline inhalation. Bars represent mean ± SD (n = 5–8 mice per condition). ∗*p* < 0.05, ∗∗*p* < 0.01, ∗∗∗*p* < 0.001, and ∗∗∗∗*p* < 0.0001 by one-way ANOVA. ALI, acute lung injury; BAL, bronchoalveolar lavage; EBA, Evans blue albumin; EC, endothelial cell; ICAM-1, intercellular adhesion molecule 1; IL, interleukin; KC, keratinocyte chemoattractant; LPS, lipopolysaccharide; MPO, myeloperoxidase; MTOR, mechanistic target of rapamycin; VE, vascular endothelial.

The critical role of endothelial MTOR in maintaining lung vascular integrity led us to compare the status of MTOR in healthy *versus* injured lungs. Lungs from mice exposed to LPS (*via* inhalation or aspiration) revealed a significant decrease in MTOR level compared to healthy lungs (Fig. 9, *A* and *B*). Similarly, the levels of VE-cadherin and VEGFR2 were also decreased in mouse lungs after LPS inhalation or aspiration (Fig. S6). Together, these findings suggest that loss of MTOR could be a common mechanism of ALI pathogenesis.

Given the role of endothelial MTOR in preserving lung vascular integrity (Figure 6, Figure 7, Figure 8), we determined whether expressing MTOR specifically in the lung endothelium is effective in protecting against ALI. To this end, we used pVE-MTOR plasmid which expresses myc-tagged MTOR from the EC-specific VE-cadherin promoter and delivered it to the lungs of mice by electroporation 48 h before LPS inhalation challenge (Fig. 9*C*). EC-targeted expression of MTOR (EC-MTOR expression) by this approach (Fig. 9*D*) reduced the lung vascular permeability and water content in LPS-treated mice compared to mice given control plasmid (Vector) (Fig. 9, *E*–*G*). Analysis of lung histology and BAL fluid from these mice also revealed a reduction in lung PMN infiltration after LPS challenge (Fig. 9, *H*–*K*). Corroborating these results, EC-MTOR expression also caused a significant decrease in the levels of proinflammatory mediators (Fig. 9, *L*–*N*). These data indicate that the prophylactic expression of MTOR in the lung endothelium is effective in protecting against LPS-induced inflammatory lung injury.

The protective effect of endothelial MTOR against lung vascular leak and inflammation led us to assess its efficacy in protecting against LPS-induced decline in lung function. LPS inhalation caused a significant decline in lung function as revealed by reduced lung compliance and increased resistance, important measures of lung expandability and alveolar pressure, respectively. Expression of MTOR in lung EC was sufficient to prevent the decline in lung function in LPS-exposed mice (Fig. 9, *O* and *P*). These data collectively establish that the loss of endothelial MTOR is a critical determinant of ALI and raise the possibility that restoring its expression in the lung endothelium could be a potential therapeutic strategy to control ALI.

### Therapeutic MTOR expression in the lung endothelium mitigates ALI and improves survival

**Figure 10:**
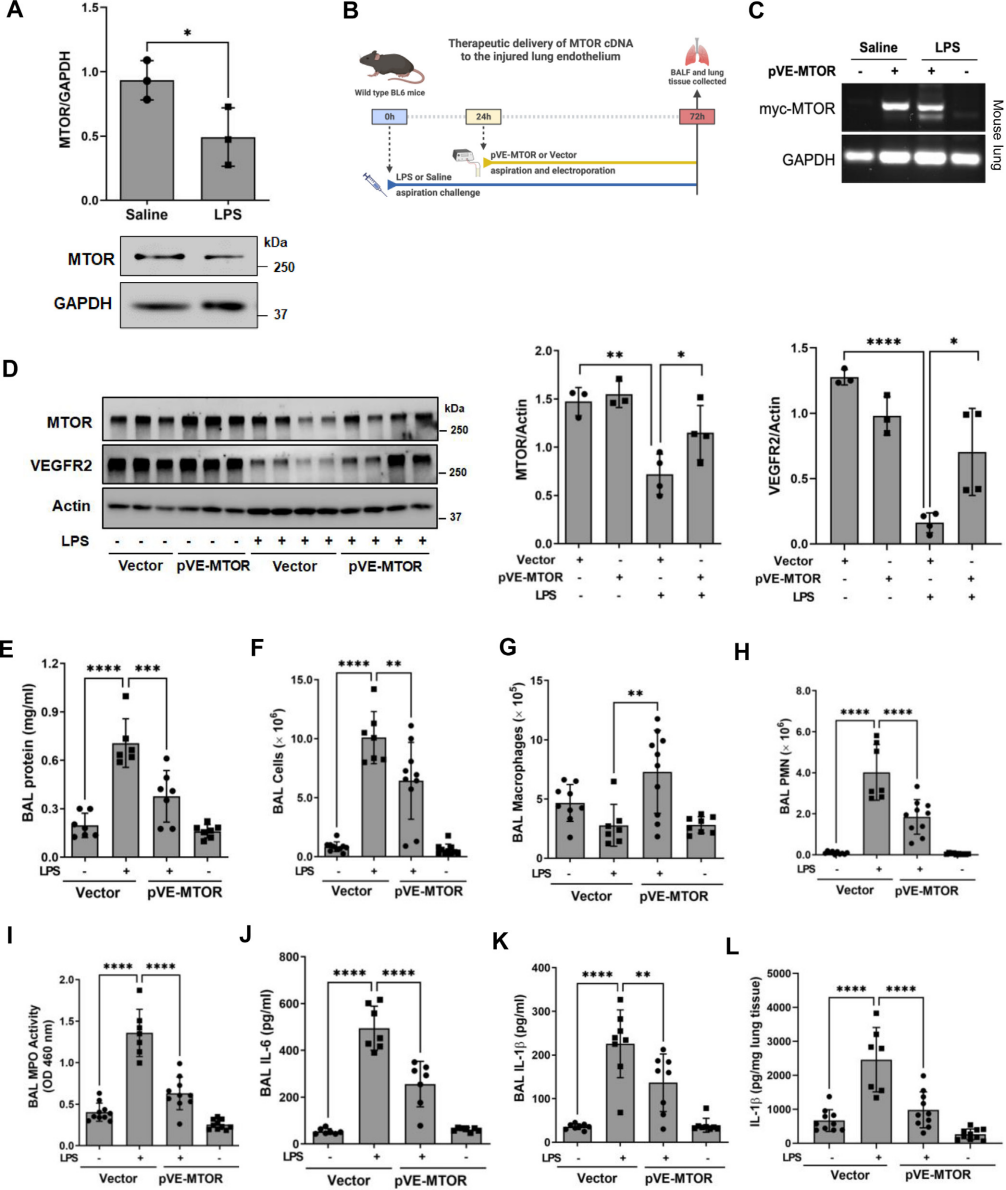
**Therapeutic expression of MTOR in the lung endothelium attenuates lung vascular leak and inflammation.***A*, WT B6 mice were challenged with saline or LPS (5 mg/kg) by aspiration. After 72 h, LMVECs were isolated from these mice as described in Experimental procedures and analyzed for MTOR and GAPDH levels. Bars represent mean ± SD (n = 3/condition). *B*, mice were challenged with saline or LPS (5 mg/kg) by aspiration and 24 h later electroporation-mediated gene transfer of pVE-MTOR in which myc-tagged MTOR complementary DNA plasmid is driven by EC-specific VE-cadherin (VE) promoter was used to selectively restore MTOR in the lung endothelium. Mice electroporated with empty vector (pRK5) were used as controls. BAL fluid and lung tissue were collected 48 h after electroporation (72 h after LPS challenge). *C*, expression of myc-tagged MTOR from the pVE-MTOR plasmid was confirmed by RT-PCR from the lungs of LPS- or saline-challenged mice with pVE-MTOR expression or Vector control. GAPDH mRNA was used to monitor loading. *D*, MTOR and VEGFR2 protein levels in the lungs of LPS- or saline-challenged mice with pVE-MTOR expression or vector control. Actin levels were used to monitor loading. Bars represent mean ± SD (n = 3–4/condition). *E*, BAL protein was determined as a measure of lung vascular leak. *F–I*, BAL cells (*F*), BAL macrophages (*G*), BAL polymorphonuclear leukocytes (PMN) (*H*), and BAL MPO activity (*I*) were determined as measures of lung PMN recruitment. *J–L*, lungs were also analyzed by ELISA to assess the levels of the proinflammatory mediators, IL-6 (*J*) and IL-1β (*K*) in BAL fluid, and IL-1β (*L*) in lung tissue. Bars represent mean ± SD (n = 7–10 mice per condition). ∗*p* < 0.05, ∗∗*p* < 0.01, ∗∗∗*p* < 0.001, and ∗∗∗∗*p* < 0.0001 by one-way ANOVA except Figure 10*D* which was analyzed by Student’s *t* test. BAL, bronchoalveolar lavage; IL, interleukin; LMVEC, lung microvascular endothelial cell; LPS, lipopolysaccharide; MPO, myeloperoxidase; MTOR, mechanistic target of rapamycin; VE, vascular endothelial; VEGFR, vascular endothelial growth factor receptor.

MTOR maintains cell proliferation, migration, and angiogenesis functions in cultured EC, all of which are critical for the resolution of vascular injury. These findings, in addition to the protective effect of prophylactic EC-MTOR expression in the lung, led us to determine the therapeutic benefit of this approach. In this treatment model, the pVE-MTOR plasmid or vector was delivered to the lung *via* electroporation 24 h after LPS aspiration (Fig. 10*B*). In contrast to the fast-acting LPS inhalation injury utilized in the prophylactic study (Fig. 9) (51), LPS aspiration causes a slower ALI progression with maximal inflammatory injury occurring around 3 days after LPS challenge (52). To determine the status of MTOR in the lung endothelium, we isolated mouse lung microvascular endothelial cells from mice exposed to LPS for 3 days. Analysis of these cells revealed a marked decrease in MTOR level (Fig. 10*A*). Consistent with this, VEGFR2 level was also reduced in these cells (Fig. S7). These results confirm that LPS challenge decreases MTOR level in the lung endothelium and is consistent with our *in vitro* data (Fig. 1).

We used this model (Fig. 10*B*) as it allows the gene transfer and expression of MTOR cDNA (Fig. 10, *C* and *D*) in mice with existing ALI to assess the therapeutic utility of this approach. BAL fluid from the lungs of LPS-treated mice with therapeutic EC-MTOR expression (LPS + pVE-MTOR) had significantly reduced BAL protein and inflammatory cell infiltration when compared to control mice (LPS + Vector), suggesting that restoring EC-MTOR expression in the lung effectively mitigates inflammatory lung injury (Fig. 10, *E*–*L*). Differential staining of BAL cells revealed an increase in macrophage and a decrease in PMN (Fig. 10, *G* and *H*), signifying a shift from a proinflammatory phenotype to a recovery phenotype. This was corroborated by a reduction in PMN activation as assessed by BAL MPO activity and attenuated levels of proinflammatory mediators (Fig. 10, *I*–*L*).

**Figure 11:**
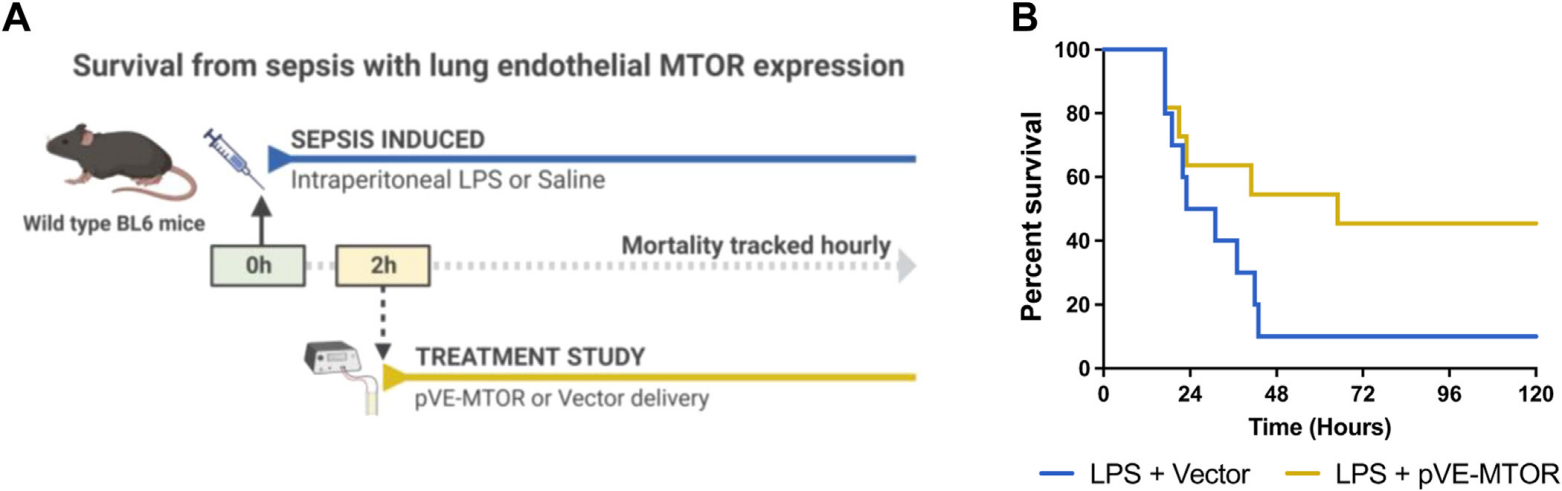
**Therapeutic expression of MTOR in the lung endothelium improves survival in mice with sepsis.***A*, therapeutic efficacy of lung EC-targeted MTOR expression against sepsis-induced mortality. Sepsis was induced by IP injection of LPS using a dose that produced 90% mortality in control mice. Two hours later, mice were electroporated with pVE-MTOR. Mice electroporated with empty vector (pRK5) were used as controls. *B*, survival rates of mice challenged with LPS and electroporated with pVE-MTOR or vector were determined over a period of 120 h. EC, endothelial cell; IP, intraperitoneal; LPS, lipopolysaccharide; MTOR, mechanistic target of rapamycin; VE, vascular endothelial.

Having demonstrated the significant protective effect of endothelial MTOR in models of direct lung injury (*i.e.*, LPS inhalation and aspiration), we assessed the capability of EC-MTOR expression in the lung to attenuate mortality from a systemic sepsis model. The rationale for using this model is that it results in high mortality compared to the above models of direct ALI. Sepsis was induced by intraperitoneal LPS injection, and the effect of therapeutic EC-MTOR expression in the lung was assessed on the mortality rate in the septic mice. Mice were first treated with LPS for 2 h and then electroporated with pVE-MTOR (Fig. 11*A*). The rationale for 2 h LPS treatment before delivering pVE-MTOR was based on a report (53) showing that LPS causes lung edema as early as 2 h which peaks at 6 h after administration. Inducing sepsis by this approach (Fig. 11*A*) resulted in ∼90% mortality within 48 h in control mice (transduced with Vector) (Fig. 11*B*). In contrast, mortality was significantly mitigated in mice with EC-MTOR expression and caused only 50% mortality 120 h after LPS (Fig. 11*B*). Notably, in the first 24 h there was no difference in mortality between the two groups which can be attributed to inadequate gene transfer/expression of the pVE-MTOR during this time. This is consistent with the reports that the delivered plasmids achieve functionally-effective expression between 16 and 72 h after gene transfer (54, 55, 56). Thus, the effective duration for LPS to cause ALI is much longer than 2 h and ALI is established before the functionally-effective expression of pVE-MTOR has taken place. It should also be noted that systemic sepsis-induced mortality was prevented by expressing MTOR in a lung EC-specific manner without affecting its expression in other organs. Collectively, these findings underline the importance of endothelial MTOR in maintaining lung vascular integrity and show that restoring its expression after injury may prove an effective therapeutic strategy against ALI.

## Discussion

Vascular homeostasis requires maintaining a critical balance between the migratory capacity of proliferative EC and the intact, semipermeable barrier function in quiescent endothelium. However, the signals governing these fundamental EC functions, particularly in the context of ALI, are poorly understood. In this study, we identify MTOR as a mechanistic link in preserving the barrier integrity and migratory/angiogenic responses in EC to maintain lung vascular normalcy. Studies in cultured EC revealed a critical role for MTOR in maintaining cell structure and function. MTOR-depleted EC had reduced barrier function, as well as slower cell proliferation and migration, and angiogenesis. MTOR-depleted EC also exhibited a marked loss of VE-cadherin and VEGFR2 mediated in part by increased autophagy in these cells. Reducing MTOR expression in mouse lung endothelium resulted in increased vascular leakage, suggesting that loss of MTOR in EC is a critical determinant of lung vascular injury. This was further evidenced by reduced MTOR level in the lungs or LMVEC from mice exposed to LPS, which raised the question of whether expressing MTOR in the lung endothelium would be protective during injury. Indeed, EC-targeted MTOR expression in the lung, either prophylactically or therapeutically, mitigated vascular permeability, inflammation, and mortality in mouse models of ALI. EC-MTOR expression was also effective in protecting against LPS-induced decline in lung function. Together, these data establish an essential role of endothelial MTOR in limiting lung vascular injury and improving lung function and survival in mice with ALI.

The functional and structural integrity of the pulmonary endothelium is essential for maintaining a semipermeable and anti-inflammatory barrier between the blood and the lung interstitium, and EC dysfunction is associated with numerous acute and chronic conditions including lung diseases (5, 6, 57). We employed *in vitro* studies to assess the function of MTOR in the quiescent endothelium. MTOR depletion revealed larger, elongated cells with increased stress fiber formation and endocytosis of cell surface VE-cadherin, leading to increased permeability in MTOR-depleted cells. These findings led us to investigate if MTOR also plays a role in controlling EC function in the proliferative phase. Indeed, MTOR-depleted cells were severely impaired in their ability to proliferate, indicating that MTOR is necessary to maintain this important EC function. Defective proliferation upon MTOR depletion was coupled with slower cell migration and impaired angiogenesis. These functional defects with *MTOR* knockdown can be attributed to loss of the angiogenic mediator VEGFR2 and its downstream signaling, rendering the cells refractory to VEGF stimulation.

We also investigated the possibility that impaired EC functions following *MTOR* silencing may be a result of these cells undergoing senescence, apoptosis, or EndMT. Although senescence has been reported to play a role in EC dysfunction (46), our results exclude this possibility as MTOR-depleted cells displayed no senescence. Similarly, there is no evidence of apoptosis or EndMT in MTOR-depleted EC; thus, it is unlikely that *MTOR* knockdown-induced EC dysfunction is driven by these processes. However, unlike senescence, apoptosis, or EndMT, *MTOR* knockdown cells exhibit increased autophagy, consistent with the role of MTOR as an inhibitor of autophagy (58). Importantly, inhibiting autophagy attenuated the loss of VE-cadherin and VEGFR2 in *MTOR* knockdown cells. Our lab has previously shown a mechanistic link between autophagy and EC inflammation and permeability (*via* autophagy proteins Beclin1 and ATG7), as well as the protective effect of autophagy inhibition in ALI (49, 59, 60). Thus, it is likely that loss of MTOR can cause endothelial dysfunction in part by promoting autophagy.

To determine if the effect of MTOR depletion on EC dysfunction can be recapitulated *in vivo* in the lung, we employed EC-targeted silencing or genetic deletion of *MTOR* in mice. Lungs or LMVEC from mice with EC-targeted MTOR deficiency each showed a marked reduction in VE-cadherin and VEGFR2 levels, consistent with our *in vitro* results. Furthermore, loss of MTOR from the endothelium resulted in increased lung vascular injury and immune cell infiltration. Surprisingly, majority of the infiltrated cells were lymphocytes, and PMN comprised only a minute fraction. This was further manifested by no appreciable increase in MPO activity and proinflammatory mediators in EC MTOR-depleted lungs. These observations are consistent with the idea that the increased immune cell influx, in addition to vascular permeability and tissue edema, is caused by endothelial barrier disruption in EC-MTOR–deficient lungs and is unlikely to be orchestrated by proinflammatory mediators whose levels remain unchanged. These data highlight an essential role of MTOR in preserving the barrier integrity and migratory capacity of the endothelium and thereby protecting against lung vascular injury.

The above finding led us to determine if loss of MTOR is a causal event in ALI. We tested this possibility by monitoring the level of MTOR in the lungs of mice exposed to LPS *via* inhalation or aspiration. In both cases, MTOR levels in the lung were markedly downregulated, consistent with our finding that EC-MTOR deficiency is sufficient to cause lung vascular permeability and tissue edema. Because MTOR levels were assessed in whole lung lysates, it is unclear which cell types of the lung, in addition to EC, are affected by this downregulation and contribute to ALI development. Our data that the levels of EC-specific proteins VE-cadherin and VEGFR2 are depleted in lungs of LPS-exposed mice (Fig. S6) indicate that the loss of MTOR in the lung is contributed, at least in part, by EC. To confirm this, we assessed the levels of MTOR in LMVEC isolated from LPS-exposed mice and HPAEC treated with LPS *in vitro* and found that MTOR levels were decreased in these cells. Similarly, LPS exposure also caused a decrease in VEGFR2 and VE-cadherin levels in these cells. These observations further support our finding that loss of MTOR in the endothelium is required and sufficient to cause lung vascular injury.

We reasoned that gene transfer and expression of MTOR cDNA selectively in the lung endothelium would mitigate the loss of MTOR by LPS and thereby allow us to definitively establish the protective role of endothelial MTOR in ALI. To test this, we expressed MTOR in lung EC before LPS injury. EC-MTOR expression by this approach ameliorated LPS-induced lung vascular injury and inflammation. Importantly, EC-MTOR expression was also effective in protecting against LPS-induced decline in lung function. These findings indicate that the loss of MTOR in the lung endothelium is a critical component of ALI progression and severity, and EC expression of MTOR *via* our gene transfer approach to replenish this loss could be an effective approach to control ALI.

Endothelial cells are ordinarily quiescent after development, but injury activates their proliferative and migratory functions to promote wound healing and oxygen delivery to damaged tissues (18, 19). We examined the possibility that the homeostatic maintenance function of MTOR in EC might promote recovery when expressed in an already-injured lung. Indeed, delivery of MTOR cDNA to the lung endothelium in mice with preexisting ALI resulted in reduced lung inflammation and injury, signifying the therapeutic potential of restoring EC-MTOR expression in the lung. As the ultimate measure of its efficacy, we tested the therapeutic potential of MTOR expression in the lung endothelium against sepsis-induced mortality. Delivering MTOR cDNA specifically to the lung EC as a therapeutic measure improved survival from sepsis. These data identify a crucial role for EC MTOR in preventing and reversing the progression of ALI and improving sepsis survival. Further study is required to determine the cause and dynamics of MTOR depletion during ALI. Identifying the timing of MTOR depletion during initial injury as well as its restoration during the resolution phase will determine a critical window of MTOR involvement in injury progression, and ideally reveal crucial intervention points for therapeutic studies.

Currently, systemic MTOR inhibitors are used clinically to treat conditions with augmented MTOR signaling, from preventing organ transplant rejection to treating viral infection and cancer; however, this study underscores the importance of protecting vascular integrity in MTOR-inhibited conditions (61, 62, 63, 64, 65). To accomplish this, future studies are required to understand the divergent roles of MTOR depending on the cellular context and the mechanisms underlying its essential functions in EC. For example, MTOR is a well-characterized oncogene in several cancers (including cancers of the lung) and its activation promotes cell proliferation, migration, and invasion, thus necessitating pharmacological MTOR inhibition to prevent this uncontrolled growth (25, 66, 67, 68). In contrast, it is critical to maintain the proliferative and migratory functions of MTOR in the endothelium to preserve vascular homeostasis. Thus, our findings raise the possibility that increased vascular permeability may be a serious complication of MTOR-targeted therapies. Indeed, patients treated with MTOR inhibitors have higher susceptibility to pneumonitis and tissue edema (69, 70).

The cell-specific role of MTOR may further limit the utility of MTOR inhibitors as a therapeutic intervention as they cannot discriminate between different cell types. This is particularly true in ALI, where MTOR has been reported to play a detrimental role in the alveolar epithelium to promote ALI (71, 72) in contrast to its function as a protective molecule in the pulmonary endothelium to limit ALI (45, 73). While the mechanisms underlying the cell-specific roles of MTOR are unclear, it is likely that the relative abundance of MTORC1 and MTORC2 differs depending on the context (endothelial *versus* epithelial) and that this could contribute to the cell-specific effects of MTOR (45, 71, 73). Thus, a therapeutic strategy that relies on cell-specific modulation of MTOR may prove a more effective approach to control ALI. Indeed, our results have demonstrated the benefit of EC-targeted MTOR expression *via* a gene transfer approach in treating ALI.

In summary, this study describes an important function of MTOR in the regulation of EC function in the quiescent and the proliferative states. MTOR depletion caused EC dysfunction through reduced barrier function, cell proliferation, and migration, and this correlated with our *in vivo* findings that depleting MTOR in the lung endothelium was sufficient to induce vascular injury. This study also highlights the downregulation of MTOR in injured lung endothelium as a key pathogenic mechanism of ALI, and EC-targeted expression of MTOR *via* gene transfer as a potential therapeutic approach to combat ALI and improve survival. Overall, our findings show a critical role of MTOR in preserving EC function and identify endothelial MTOR as an effective therapeutic target in ALI.

## Experimental procedures

### Reagents

Antibodies to MTOR (2983S), VEGFR2 (9698S), phospho-VEGFR2^Y1175^ (3770S), phospho-VEGFR2^Y1059^ (3817S), phospho-ENOS^S1177^ (9571S), eNOS (32027S), phospho-AKT^S473^ (4060S), AKT (2938S), Snail (3879T), LC3A/B (12741T), and N-cadherin (13116T) were obtained from Cell Signaling Technology. Antibodies to VE-cadherin were purchased from BD Biosciences (BD555661), Invitrogen (36-1900), and Santa Cruz Biotechnology (SC-6458). Anti-PCNA (SC-56), anti-β-actin (SC-47778), anti-GAPDH (SC-32233), p62 (SC-28359), and p38 (SC-535) antibodies were from Santa Cruz Biotechnology. Antibodies to phospho-ERK^Y204^ (AP0235) and ERK (A19561) were purchased from AbClonal and α-smooth muscle actin (MAB1420) from R&D systems. Click-iT EdU assay kit and bafilomycin A (B1793) was obtained from Invitrogen. *In vitro* Vascular Permeability Assay kit and Cellular Senescence Assay Kit were from Sigma-Aldrich. Plasmid maxi kit and RNeasy mini kit were from Qiagen Inc. All other materials were purchased from Thermo Fisher Scientific.

### Endothelial cell culture

Primary HPAECs were obtained from Lonza and cultured as described (43). Cells were grown in gelatin-coated flasks in endothelial basal medium 2 (EBM2) containing 10% fetal bovine serum and bullet kit additives (Bio Whittaker) in a humidified 37 °C incubator supplemented with 5% CO_2_. For LPS treatment, cells were incubated in 2% fetal bovine serum-containing EBM2 or Dulbecco's modified Eagle's medium for 12 h prior to stimulation with LPS (5 μg/ml) (74). HPAEC between passages 3 and 7 were used. LMVECs from mice were isolated as described (50). Briefly, lungs isolated from mice were minced, digested with collagenase at 37 °C for 60 min, and centrifuged at 1000*g*. The cells were resuspended and then incubated with anti-CD31–coated Dynabeads for 2 h. Cells were then magnetically sorted and cultured in growth medium EBM2 containing endothelial growth supplement and 20% fetal bovine serum (74).

### siRNA-mediated depletion of MTOR

siRNA specific for human MTOR (siMTOR) was obtained from Cell Signaling Technology, and DharmaFect1 siRNA Transfection Reagent and a nonspecific control siRNA (siControl) were obtained from Horizon Discovery. HPAECs were transfected with siControl or siMTOR according to DharmaFect1 transfection protocol (75). Briefly, 50 to 100 nM siRNA and DharmFect1 reagent were mixed and then incubated with subconfluent cells for 24 h, after which fresh complete EBM2 media was added. Cells were utilized in experiments after 48 h of transfection unless otherwise indicated.

### Cell lysis and immunoblotting

After treatment, HPAECs were lysed in radioimmunoprecipitation buffer (Thermo Fisher Scientific) supplemented with EDTA-free protease inhibitor cocktail tablet (Roche Diagnostics) as described (76). Cell lysates were resolved on SDS-PAGE and transferred onto nitrocellulose membranes. Membranes were incubated with 5% (w/v) nonfat dry milk in Tris-buffered saline + Tween 20 [10 mM Tris (pH 8.0), 150 mM NaCl, and 0.05% Tween 20] for 1 h at room temperature, then detected with antibodies and developed with SuperSignal West Femto Substrate (Thermo Fisher Scientific), and visualized using Chemi-Doc Imaging System (Bio-Rad Laboratories Inc). Densitometric analysis of band intensity was performed using “Measure” tool in Fiji (https://imagej.net) (77).

### Immunofluorescence

Confluent HPAECs were grown on gelatin-coated glass coverslips and subjected to immunofluorescent staining as described (59). Fixed cells were incubated with Alexa Fluor 594-phalloidin (Invitrogen) for 20 min at room temperature to detect F-actin filaments. To visualize cell surface VE-cadherin, fixed cells were incubated with VE-cadherin antibody (BD Biosciences) and anti-mouse Alexa Fluor 488 secondary antibody (Invitrogen). Whole cell VE-cadherin (*i.e.*, intracellular and cell surface) was detected by pre-incubation in PBS-triton before antibody staining to permeabilize the cell membrane. To determine the percentage of cells with disrupted AJs, cells with discontinuous cell surface VE-cadherin staining were counted and normalized to the total number of cells (28, 29, 30, 31, 32, 33, 34, 35, 36, 37, 38, 39, 40, 41, 42, 43, 44, 45, 46, 47, 48, 49, 50, 51, 52, 53, 54, 55, 56, 57, 58, 59, 60, 61, 62, 63, 64) in the field (59). The percentage of cells with discontinuous cell surface VE-cadherin staining was averaged and presented as bar graphs. To visualize the internalized VE-cadherin, live ECs were stained with an anti-VE-cadherin extracellular domain antibody in the presence of 100 μM chloroquine at 37 °C for 4 h (78). The labeled cells were then briefly subjected to mild acid buffer (2 mM glycine-PBS, pH 2.0, 2 min) and washed twice with PBS. 4',6-diamidino-2-phenylindole (Thermo Fisher Scientific) was used to stain nuclei. Coverslips were mounted on glass slides using Fluoromount mounting media (Southern Biotech) and images were acquired using a Leica TCS SP5 laser or Leica Stellaris 5 confocal microscope (Leica Microsystems).

### Cell proliferation assay

After transfection with siControl or siMTOR for 24 h, cells were replated at a subconfluent density (∼20,000 cells/well) in gelatin-coated 12-well plates. Each day up to 6 days after plating, three wells per treatment were trypsinized, and cells were counted to assess cell proliferation. The average cell number per day for each treatment was graphed.

### Assessment of endothelial barrier integrity by TEER

TEER was measured across confluent monolayers using the Electrical Cell-Substrate Impedance Sensing system (Applied Biophysics) as described (49, 79). Briefly, HPAECs were grown on gelatin-coated gold microelectrodes, and TEER was measured after confluency to determine baseline resistance. To assess cell migration, the Electrical Cell-Substrate Impedance Sensing system generated a 250 μm wound in the confluent monolayer by pulsing with 1400 μAmps for 15 s. TEER was measured over a period of 16 h and normalized to baseline resistance. The rate of cell migration was calculated using the formula (TEER_initial_ − TEER_final_)/(hours elapsed), and the rate was graphed.

### Scratch assay

Confluent HPAEC monolayers grown in gelatin-coated 12-well plates were wounded by making a single scratch along the well diameter with a 200 μl pipet tip. Media was aspirated to remove dislodged cells and replaced with fresh complete media or media supplemented with VEGF_165_ (50 ng/ml, 293-VE, R&D Systems) where indicated. Cells were incubated at 37 °C with 5% CO_2_ to allow for recovery, and images were acquired at the indicated time points using inverted microscope. The wound width was determined at four intervals per scratch using the “Measure” tool in Fiji, and the average width (in μm) for each condition was determined (77). The rate of cell migration was calculated using the formula (width_initial_ − width_final_)/(hours elapsed), and the rate was graphed.

### Matrigel tube formation assay

Matrigel Basement Membrane Matrix (Corning Life Sciences) was used to perform the tube formation assay as described (80). HPAECs were transfected with appropriate siRNA. After 48 h, equal numbers of HPAECs were seeded on Matrigel at subconfluent density and imaged 6 h after incubation. Images were analyzed using the Angiogenesis Analyzer plugin for Fiji (77, 81). Briefly, *junctions* define branching points, and *segments* are lines connected to two junctions. The *branching length* is calculated by the sum of the length of branching trees.

### Generation of mice with lung EC-restricted MTOR depletion/deletion

To selectively *deplete* MTOR in mouse lung EC, WT C57BL/6J mice (Jackson Laboratory) were transduced with plasmids expressing MTOR shMTOR (shMTOR) or nontargeting shRNA (shControl) *via* cationic liposomes. Cationic liposome-mediated gene delivery is a well-accepted method for transferring a gene of interest selectively to the lung endothelium irrespective of the promoter used (82, 83, 84, 85, 86). Cationic liposomes were prepared as described (86). shMTOR plasmid [∼50 μg (1 μg/μl) DNA] was gently mixed with cationic liposomes (100 μl), and the shMTOR plasmid/cationic liposome complexes were delivered by retro-orbital vein injection. After 3 days, lungs from these mice were isolated and analyzed for markers of inflammation and injury. The expression level of MTOR in the lung or LMVEC isolated from these mice were determined by immunoblotting.

To further validate the findings made using the above approaches, the CRE-lox system was utilized for EC-restricted deletion of the *MTOR* gene in mice. Agouti *Mtor*^*fl/fl*^ (Allele: *Mtor*^tm1.1Gcon^) mice were kindly provided by Dr Gianluigi Condorelli (University of California San Diego) and C57BL/6J *Mtor*^*fl/fl*^ mice were generated by breeding these mice with wild type C57BL/6J mice at the University of Rochester by the vivarium staff (40). The C57BL/6J *Mtor*^*fl/+*^ mice were backcrossed to C57BL/6 for several generations (>10) and subsequently interbred to obtain C57BL/6J *Mtor*^*fl/fl*^ mice. These mice (C57BL/6J *Mtor*^*fl/fl*^) were bred with mice constitutively expressing Cre under the VE-cadherin promoter (*VE-cadherin*^*Cre*^ mice, strain: B6.FVB-Tg(*Cdh5^C^^re^*)7Mlia/J, Jackson Laboratory [obtained from Dr Jun-Ichi Abe]) to generate mice with heterozygous EC MTOR deletion (*VE-cadherin*^*C*^^*re*^^*+*^/*Mtor*^*fl/+*^ [EC-*Mtor*^*+/−*^]) (40, 87). Lungs from EC-*Mtor*^*+/−*^ or control (*Mtor*^*fl/fl*^) mice were isolated and analyzed for markers of inflammation and injury. Genotypes of mice were authenticated by PCR analysis using mouse tail DNA as described (40). DNA extraction and PCR analysis was performed using REDExtract-N-Amp Tissue PCR Kit (Sigma-Aldrich) and primers targeting *Cre* (F: CCAAAATTTGCCTGCATTACCGGTCGATGC, R: ATCCAGGTTACGGATATAGT) and *Mtor* floxed gene (F: GCTCACTGTACTCTGTCTGCACTTG, R: GAAATAGCACGCATTTCTACTTG), obtained from Integrated DNA Technologies. In some experiments, we used our recently described gene transfer-based approach (50) to delete *Mtor* in the lung endothelium. Briefly, *Mtor*^*fl/fl*^ mice were delivered with pRK5 (empty vector) or pVECad-CRE in which Cre is driven by EC-specific VE-cadherin promoter *via* cationic liposomes as described above. After ∼10 days, lungs from these mice were isolated and analyzed for lung vascular leak and tissue edema.

### Electroporation-mediated gene transfer of MTOR cDNA in the lung endothelium

Wild type C57BL/6J mice (Jackson Laboratory) were anesthetized by isofluorane inhalation (2–3% with O_2_) and EC-specific VE-cadherin promoter-driven MTOR cDNA plasmid (pVE-MTOR) or empty vector (pRK5) (100 μg plasmid DNA in 50 μl PBS per mouse) was aspirated for 1 min and then electroporated. To this end, pediatric pacemaker electrodes (Medtronic) were placed on both sides of the chest and 8 to 10 ms square wave pulses at field strength of 200 V cm^−1^ were immediately applied with an ECM830 electroporator (BTX, Harvard Apparatus) (88, 89). After the experiment, successful gene transfer and expression was confirmed by RT-PCR to detect mRNA for *Myc* tag (F: GGAACAAAAGCTCATTAGCGAGG, R: TGATGGCCAGCTCACGAGAACGAG) and *Gapdh* (F: CCATCACCATCTTCCAGGAG, R: CCTGCTTCACCACCTTCTTG) in the lungs of mice. Plasmid maxi kit and RNeasy mini kit were from Qiagen Inc. iScript cDNA Synthesis Kit and SsoAdvanced Universal SYBR Green Supermix were purchased from Bio-Rad Laboratories Inc. DNA primers were obtained from Integrated DNA Technologies.

### Murine models of ALI

*Escherichia coli* LPS (Sigma-Aldrich) was utilized in three murine models of ALI. In the inhalation model, mice were exposed to aerosol of LPS (1.0 mg/ml in 6 ml PBS; *E. Coli* 0111:B4) or saline alone for 30 min as previously described, and mice were euthanized after 18 to 20 h (79). In some experiments, LPS was delivered *via* aspiration. Briefly, mice were anesthetized with isofluorane (2–3% with O_2_) and LPS (5.0 mg/kg in 50 μl PBS; *E. Coli* O55:B5; Sigma-Aldrich) was delivered to mouse lungs by oropharyngeal aspiration and mice were euthanized 3 days later (90). Lungs from both LPS inhalation and aspiration mice were analyzed for markers of lung inflammation and injury. In mortality experiments, sepsis was induced by intraperitoneal injection of LPS (*E. Coli* 0111:B4), and survival was monitored for 120 h (78, 86). In both experiments, mice were challenged with LPS using doses that produced LD90 mortality rate in control mice.

### Evaluation of lung inflammation and injury

BAL and lung tissue were collected from mice as previously described (79). Mouse lung homogenates were prepared in ready-to-use radioimmunoprecipitation buffer supplemented with protease cocktail inhibitor (Sigma-Aldrich) as previously described (79, 91, 92). Lung homogenates were analyzed by immunoblotting and detected with anti-VE-cadherin, anti-VEGFR2, anti-MTOR, and anti-β-actin antibodies. The levels of monocyte chemoattractant protein-1, keratinocyte chemoattractant, interleukin-6, interleukin-1 beta, and intercellular adhesion molecule 1, in lung homogenate or BAL fluid were determined using ELISA kits from R&D Systems, and BAL Albumin was determined by ELISA kit from Alpco. Protein content of BAL fluid was assessed using the Pierce BCA Protein Assay Kit (Thermo Fisher Scientific), and PMN recruitment in the lung was determined by myeloperoxidase activity in BAL fluid as previously described (79, 92, 93). Differential staining of BAL cells was performed using RAL Diff-Quik (Siemens Medical Solutions). Evans blue-conjugated albumin leakage and lung wet-to-dry weight ratio were measured in mice that were not subjected to BAL as described elsewhere (49).

### Histology

Lungs were collected and fixed in 10% formalin, and paraffin-embedded sections (5 μm) were mounted on slides. Lung sections were stained with H&E for histological analysis and imaged with a Nikon Eclipse E400 microscope at 400x magnification (49, 92).

### Lung function studies

Dynamic lung compliance and lung resistance were measured in live ventilated mice using a whole-body plethysmograph (BUXCO Electronics) and a Harvard rodent ventilator (Harvard Apparatus) as previously described (93, 94). Dynamic lung compliance was normalized to the peak body weight of each animal. Data were collected and analyzed using the Biosystems XA software package (https://datasci.com) (BUXCO Electronics).

### Statistics

Student’s *t* test was performed to analyze the difference between two groups, and one-way ANOVA with Tukey’s multiple comparisons test was used to analyze multiple groups. Statistical analyses were performed using GraphPad Prism version 9.4.1 for Mac OS X (GraphPad Software, https://graphpad.com). Data is presented as mean ± SD, and a *p* value < 0.05 was considered statistically significant.

### Study approval

All animal care and treatment procedures were approved by the University of Rochester Committee on Animal Resources and performed in accordance with National Institutes of Health guidelines. Experiments were conducted on male mice except where indicated.

## Data availability

All data are available contained in the main text and the supporting information.

## Supporting information

This article contains supporting information.

## Conflict of interest

The authors declare that they have no conflicts of interest with the contents of this article.
