# Supplementary material for: MTOR maintains endothelial cell integrity to limit lung vascular injury
Source: J Biol Chem. 2024 Nov 6;300(12):107952. doi: 10.1016/j.jbc.2024.107952 (PMC11664419; doi:10.1016/j.jbc.2024.107952)
Supplement: Supporting information [file mmc1.pdf]

SUPPORTING FIGURES

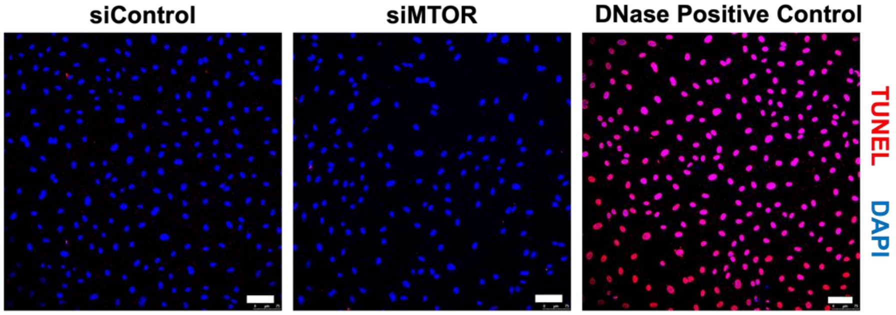

Figure S1. MTOR depletion does not induce apoptosis. HPAEC were plated on glass coverslips and transfected with siControl or siMTOR. Cells were fixed after 48 h transfection and TUNEL staining was performed to mark the nuclei of apoptotic cells (red), and DAPI (blue) was used to mark all nuclei. DNase treatment was performed as a positive control.

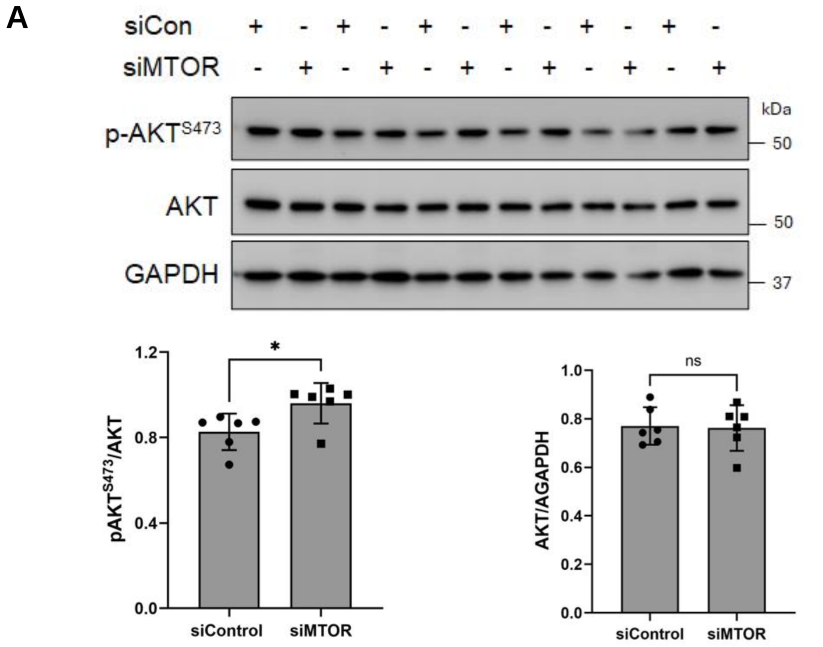

Fig S2A Effect of MTOR depletion on AKT phosphorylation. HPAEC were transfected with siControl or siMTOR for 48 h. Cells were lysed and immunoblotted for phsopho-AKT^{S473}, AKT and GAPDH levels. Data are mean ± SD (n = 6). *p < 0.05 by Student's T-test.

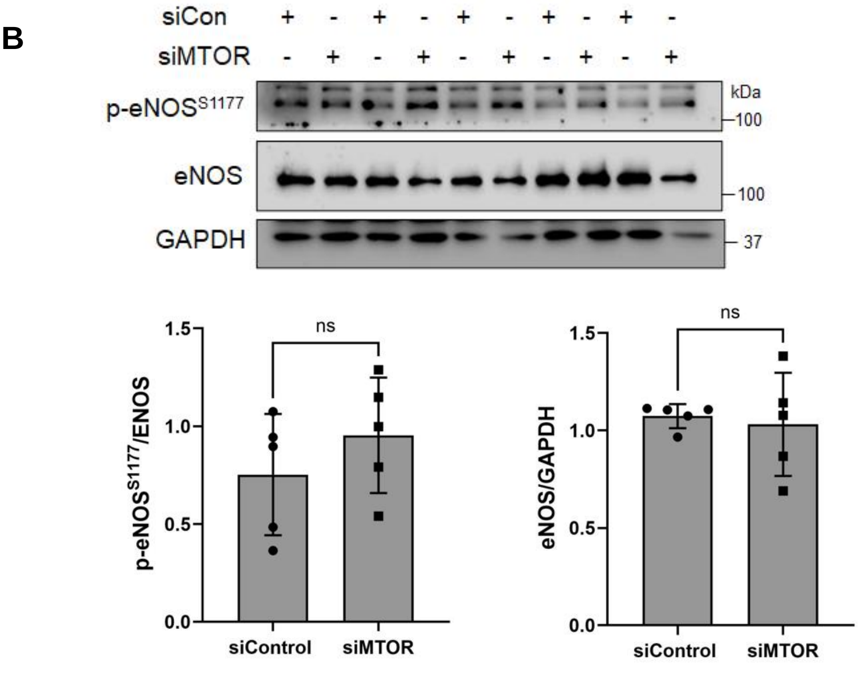

Fig S2B Effect of MTOR depletion on eNOS phosphorylation. HPAEC were transfected with siControl or siMTOR for 48 h. Cells were lysed and immunoblotted for phsopho-eNOS^{S1177}, eNOS and GAPDH levels. Data are mean ± SD (n = 5).

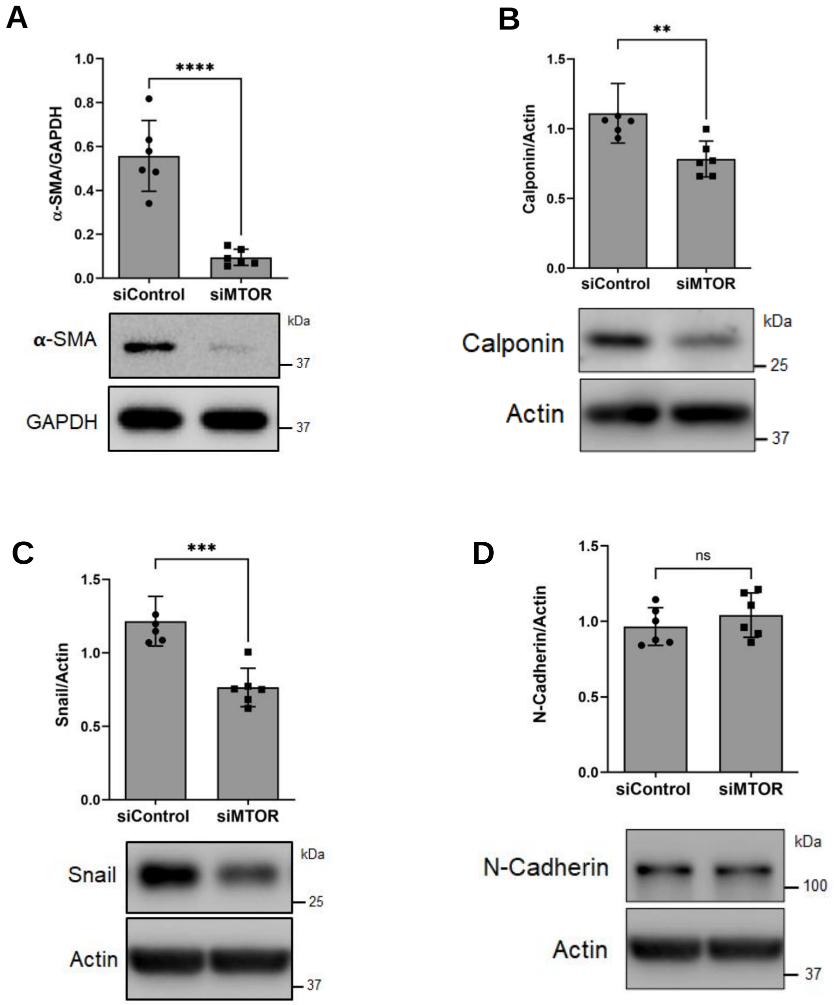

Fig S3. Effect of MTOR depletion on EndMT markers in EC. HPAEC were transfected with siControl or siMTOR for 48 h. Cell lysates were analyzed by Western blot to determine ***(A)*** α-SMA, ***(B)*** Calponin, ***(C)*** Snail, and ***(D)*** N-Cadherin. GAPDH or Actin levels were used as loading control. Bars indicate mean ± SD (n = 6 per condition). ** $p < 0.01$, *** $p < 0.001$, **** $p < 0.0001$ by Student's T-test.

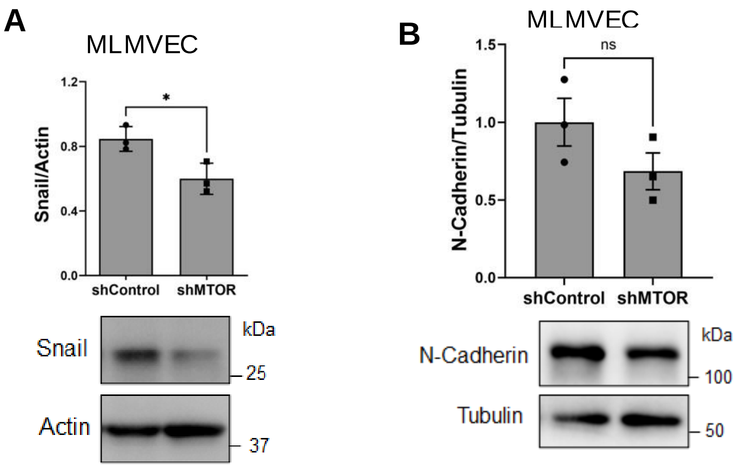

Fig S4. Effect of MTOR knockdown in the lung endothelium on EndMT markers. WT mice were transduced with shControl or shMTOR plasmid via cationic liposomes. After 72 h, lung microvascular endothelial cells (LMVEC) were isolated from these mice and cell lysates were analyzed for ***(A)*** Snail or ***(B)*** N-Cadherin level by immunoblotting. Actin ***(A)*** or tubulin ***(B)*** levels were used to monitor loading. Bars indicate mean ± SD (n = 3 per condition). * $p < 0.05$, by Student's T-test.

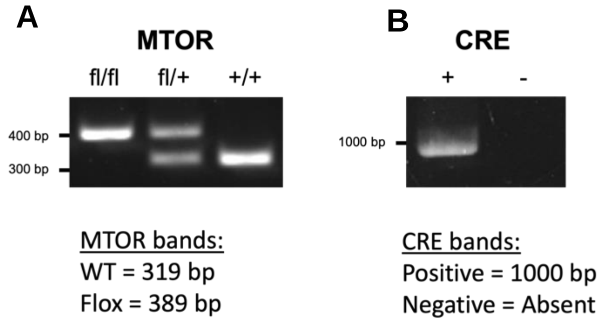

Figure S5. Genotyping of VE-CadherinCRE^{+}/MTOR$^{fl/+}$ (EC-MTOR$^{+/-}$) mice. DNA extracted from mouse tail clippings was amplified by PCR to detect ***(A)*** *Mtor* floxed allele or ***(B)*** *Cre* allele.

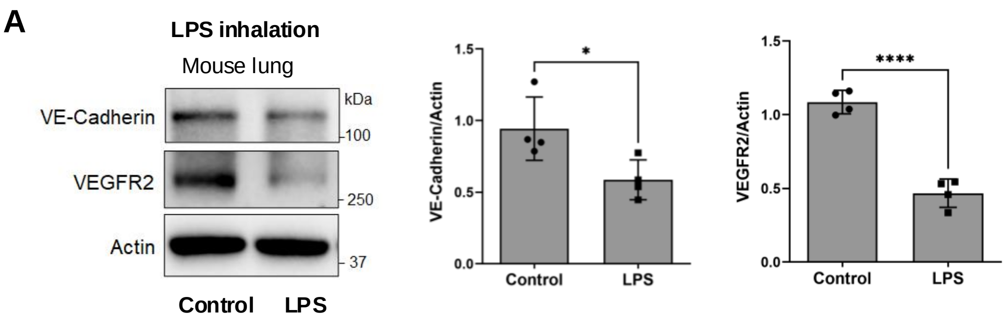

Figure S6A. VE-Cadherin and VEGFR2 levels are decreased in the lungs of mice exposed to LPS inhalation. WT mice were challenged with LPS (1 mg/ml in 6 ml saline) via inhalation. After 20 h, lungs were extracted, lysed, and analyzed by Western blot for VE-Cadherin or VEGFR2 levels. Actin was used to monitor loading. Bars represent mean ± SD (n=4 mice/condition). * $p < 0.05$, **** $p < 0.0001$ by Student's T-test.

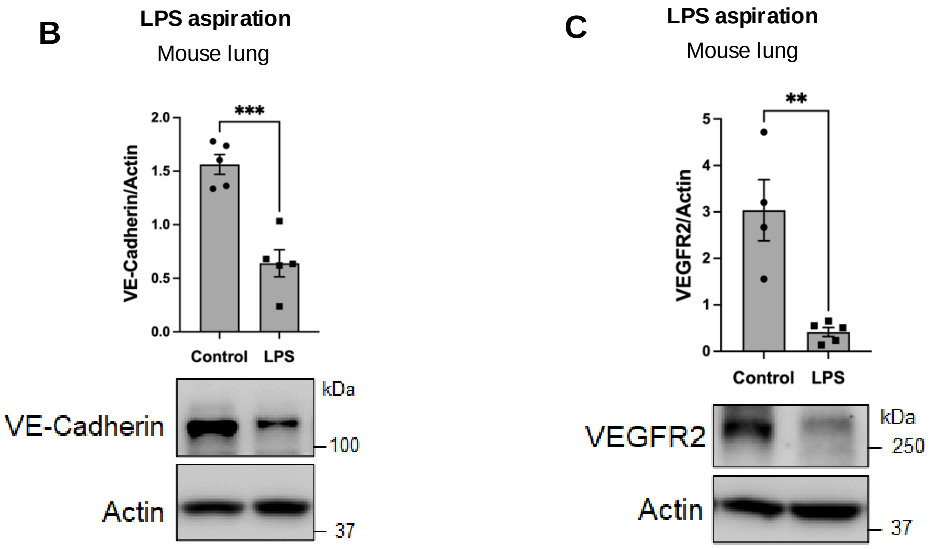

Figure S6 B&C. VE-Cadherin and VEGFR2 levels are decreased in the lungs of mice exposed to LPS aspiration. WT mice exposed to LPS (5 mg/kg) via aspiration for 72 h and lungs were extracted, lysed, and analyzed by Western blot for ***(B)*** VE-Cadherin or ***(C)*** VEGFR2 levels. Actin was used to monitor loading. Bars represent mean ± SD (n=4-5 mice/condition). ** $p < 0.01$, *** $p < 0.001$ by Student's T-test.

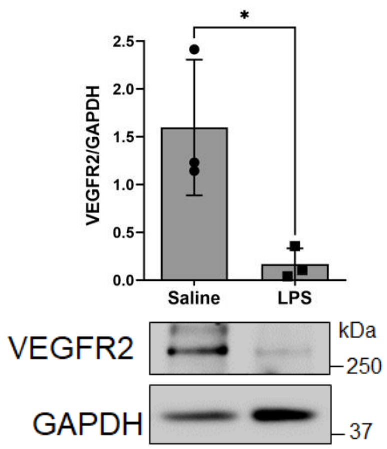

Figure S7. LPS exposure reduces VEGFR2 level in the lung endothelium. WT mice were challenged with saline or LPS (5 mg/kg) by aspiration. After 72 h, LMVEC were isolated from these mice and analyzed for VEGFR2 and GAPDH levels. Bars represent mean ± SD (n = 3/condition). GAPDH was used to monitor loading. Bars represent mean ± SD (n = 3 per group). * $p < 0.05$ by Student's T-test.
